# Dormant tumors circumvent tumor-specific adaptive immunity by establishing a Treg-dominated niche via DKK3

**DOI:** 10.1172/jci.insight.174458

**Published:** 2023-11-22

**Authors:** Timothy N. Trotter, Carina E. Dagotto, Delila Serra, Tao Wang, Xiao Yang, Chaitanya R. Acharya, Junping Wei, Gangjun Lei, H. Kim Lyerly, Zachary C. Hartman

**Affiliations:** 1 Department of Surgery, and; 2 Department of Pathology/Integrative Immunobiology, Duke University, Durham, North Carolina, USA.

**Keywords:** Immunology, Oncology, Breast cancer, Cancer immunotherapy

## Abstract

Approximately 30% of breast cancer survivors deemed free of disease will experience locoregional or metastatic recurrence even up to 30 years after initial diagnosis, yet how residual/dormant tumor cells escape immunity elicited by the primary tumor remains unclear. We demonstrate that intrinsically dormant tumor cells are indeed recognized and lysed by antigen-specific T cells in vitro and elicit robust immune responses in vivo. However, despite close proximity to CD8^+^ killer T cells, dormant tumor cells themselves support early accumulation of protective FoxP3^+^ T regulatory cells (Tregs), which can be targeted to reduce tumor burden. These intrinsically dormant tumor cells maintain a hybrid epithelial/mesenchymal state that is associated with immune dysfunction, and we find that the tumor-derived, stem cell/basal cell protein Dickkopf WNT signaling pathway inhibitor 3 (DKK3) is critical for Treg inhibition of CD8^+^ T cells. We also demonstrate that DKK3 promotes immune-mediated progression of proliferative tumors and is significantly associated with poor survival and immunosuppression in human breast cancers. Together, these findings reveal that latent tumors can use fundamental mechanisms of tolerance to alter the T cell microenvironment and subvert immune detection. Thus, targeting these pathways, such as DKK3, may help render dormant tumors susceptible to immunotherapies.

## Graphical abstract

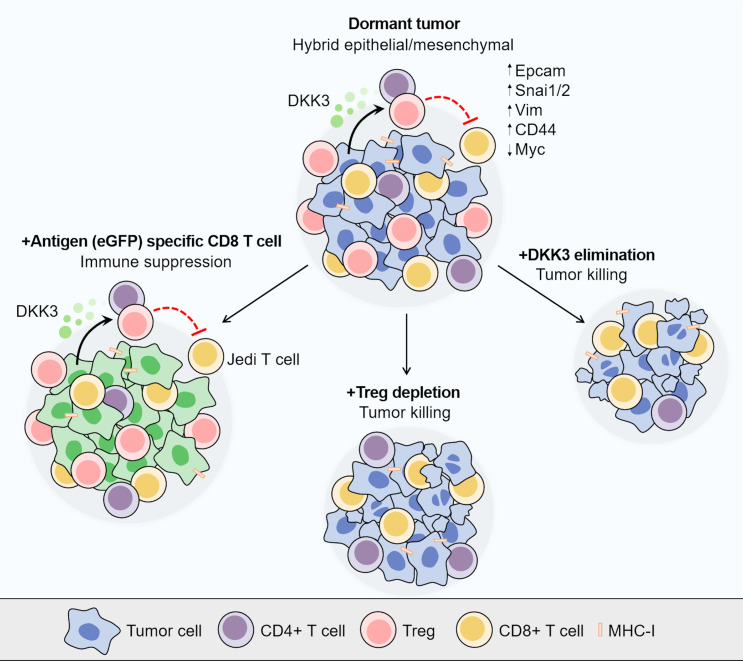

## Introduction

Multiple therapies now exist to treat different molecular subtypes of breast cancer (BC), leading to steady improvement in survival over the past 2 decades (1). Despite these successes, many survivors (approximately 30%) will eventually experience locoregional or metastatic recurrence, even when there was no clinical evidence of disease after initial therapy (2, 3). Among solid tumors, BC has a propensity for delayed relapse, with distinct patterns of recurrence based on subtype. Those with triple-negative BC (TNBC), defined by lack of the estrogen receptor (ER), progesterone receptor (PR), and human epidermal growth factor receptor 2 (HER2), are particularly at risk of distant recurrence, with a shorter window than other subtypes (33.9% vs. 20.4%; 2.6 vs. 5 years, respectively) (4). In contrast, ER^+^ tumors can recur up to decades after treatment of the primary tumor and have a consistent risk of recurrence over time (4, 5). Regardless, the time between remission and relapse offers a critical window to eliminate residual tumor cells before developing resistance mechanisms that make recurrent tumors exceedingly challenging to treat.

This phenomenon of delayed relapse, often described generally as tumor dormancy, is largely attributable to residual tumor cells that enter a state of quiescence or minimal proliferation until some other condition for growth is attained (6). To date, multiple mechanisms help explain how these cells enter and exit quiescence. However, relatively little is known regarding their function during the intervening period. Though they are mostly nonproliferative, dormant cancer cells actively communicate with the local stroma to alter the microenvironment and support their own survival (7, 8). Thus, understanding the intrinsic biology of residual, dormant tumor cells is necessary if attempting to eliminate them before recurrence.

The advent of immunotherapy has highlighted the role of immune cells in an evolving tumor, even during dormancy. Organ transplantation provided early evidence that the immune system prevents tumor outgrowth when occult tumors from donated organs began growing in the context of immunosuppressed recipients (9). Furthermore, direct evidence that tumor-specific T cells prevent metastatic outgrowth was also recently described in BC (10, 11), although how residual, dormant tumor cells are not eliminated remains in question. A potential explanation may be that fundamental, developmental mechanisms of tolerance, such as those present at the maternal-fetal interface or during mammary involution, are coopted to prevent complete tumor elimination (12). While unclear, if immunotherapy is to provide a lasting cure for patients, these overarching processes must be better understood.

Herein, we demonstrate that dormant tumors are recognizable by T cells, but manage to persist in the presence of activated tumor antigen–specific CD8^+^ T cells. Our studies reveal that dormancy-competent tumor cells maintain a hybrid epithelial/mesenchymal (E/M) or mammary progenitor-like state in vitro and in vivo. To survive long term, these cells induce a CD4^+^/Treg–shifted T cell profile via secreted factors and we identify the Wnt pathway antagonist, Dickkopf WNT signaling pathway inhibitor 3 (DKK3), as a crucial mediator of this effect. Together, the data presented here provide insight into protective barriers that prevent elimination of dormant tumors and lay the foundation for new targets that may be combined with current immunotherapies to provide more durable responses for patients.

## Results

### D2.1 dormancy is immune independent.

**Figure 1:**
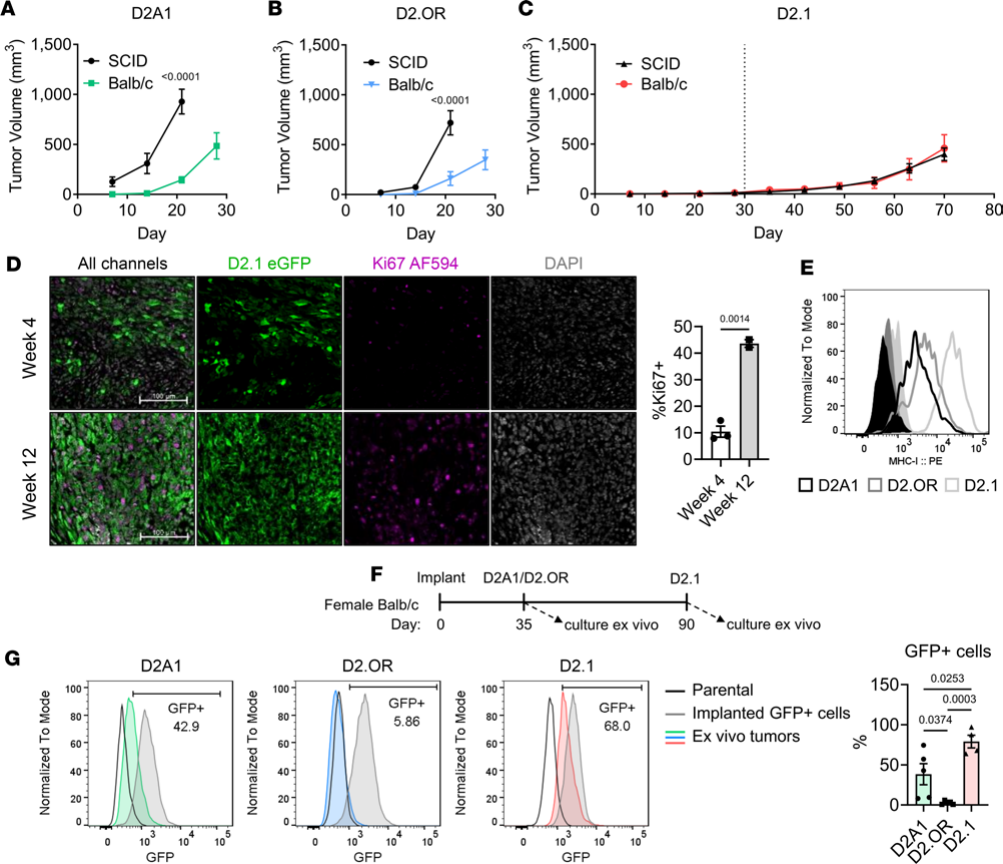
The adaptive immune system does not impact tumor dormancy or long-term persistence in mammary D2.1 tumors. (**A**) Tumor growth in the mammary fat pad (MFP) of 1 × 10^6^ parental D2A1 cells in BALB/c or SCID-beige mice (*n* = 5/group). (**B**) D2.OR tumor growth of 1 × 10^6^ parental cells in BALB/c or SCID-beige mice (*n* = 5/group). Comparisons shown are at time of first euthanasia and were performed by Šídák’s 2-way ANOVA (**A** and **B**). (**C**) Growth of parental D2.1 cells (1 × 10^6^) implanted into the MFP of BALB/c (*n* = 5) or SCID-beige (*n* = 8) mice. (**D**) Representative images (left) or quantification (right) of Ki67^+^eGFP^+^ tumor cells after MFP implantation of 1 × 10^6^ D2.1-eGFP cells in BALB/c mice and collection after 4 or 12 weeks. Tumor sections were stained for nuclei (DAPI, pseudocolor gray), eGFP (green), or Ki67 (purple). Scale bars: 100 μm. Statistical comparison was by 2-tailed *t* test. (**E**) Flow cytometric analysis of MHC-I surface expression on cultured D2A1, D2.OR, and D2.1 cells. (**F**) D2A1-eGFP, D2.OR-eGFP, or D2.1-eGFP single-cell clones (*n* = 5/group) were implanted (1 × 10^6^ cells/mouse) into the MFP of female BALB/c mice. Tumors were resected at the indicated time point, digested, and cultured ex vivo for a pure tumor cell population. (**G**) Left: Representative flow plots for eGFP expression of ex vivo D2A1, D2.OR, or D2.1 tumors compared to parental (no eGFP) cells or respective single-cell clones on the day of injection. Right: Quantification of the percentage of cultured tumor cells that maintained eGFP expression after in vivo selection. Statistical comparisons were performed by 1-way ANOVA with Tukey’s post hoc correction. Data are presented as mean ± SEM.

The D2 series of cells, consisting of D2A1, D2.OR, and D2.1 cell lines that arose from D2 hyperplastic alveolar nodules in female BALB/c mice, were used to investigate immunity in tumor dormancy (13, 14). Each cell line is reported to equally extravasate into the lung parenchyma, but only D2A1 rapidly proliferates, while D2.OR and D2.1 remain dormant in the metastatic setting (13, 15, 16). Tumor behavior in the presence or absence of adaptive immunity was first determined by implanting these cells in the mammary fat pad (MFP) of BALB/c (immunocompetent) or SCID-beige (deficient in T and B cells and defective NK cells) mice. Both D2A1 and D2.OR tumor growth was significantly delayed in the presence of adaptive immunity (Figure 1, A and B). In contrast, D2.1 tumors were unaffected by T, B, and NK cells (Figure 1C). However, when cultured in vitro, D2A1 and D2.1 cells proliferated at approximately the same rate until confluence, at which point D2.1 cells became contact inhibited, resulting in slowed growth (Supplemental Figure 1, A and B; supplemental material available online with this article; https://doi.org/10.1172/jci.insight.174458DS1). To validate the dormant phenotype, D2.1 cells were stably transduced with eGFP by lentiviral vectors and proliferation was assessed in resultant tumors by Ki67 immunofluorescence (IF) at 4 or 12 weeks after MFP implantation in BALB/c animals. Staining revealed that eGFP^+^ D2.1 tumor cells were minimally positive for Ki67 at 4 weeks, but had significantly elevated Ki67 expression by 12 weeks (Figure 1D). Co-injection of a 1:1 mix of D2A1 and D2.1 cells also resulted in approximately equivalent tumor growth as D2A1 cells alone, suggesting dormant cells do not induce quiescence in otherwise proliferative cells (Supplemental Figure 1C). Collectively, these results demonstrate that D2.1 tumor cells exist as a separate, intrinsically dormant population, unaffected by immune status, that have the eventual capacity to develop into proliferative tumors.

### Dormant tumors are immunologically protected from infiltrating T cells.

Because D2.1 tumor persistence through the dormant phase was unaffected by adaptive immunity, variation in cell surface expression of major histocompatibility complex class I (MHC-I), programmed death-ligand 1 (PD-L1), and CD47 were investigated as potential mechanisms for dormancy-mediated immune evasion (17, 18). Notably, MHC-I (Figure 1E and Supplemental Figure 1D), PD-L1, and CD47 (Supplemental Figure 1, E and F) are expressed similarly on D2A1, D2.OR, and D2.1 cells, suggesting that these are unlikely to mediate differences in immune resistance. As all D2 lines express comparable cell surface MHC-I, loss of eGFP expression after in vivo implantation was used as a readout for antigen-specific immune selection (Figure 1F) (19). While many D2A1 tumor cells resisted eGFP-specific selection, D2.OR cells displayed the highest sensitivity, with almost complete loss of eGFP (Figure 1G). In contrast, the majority of D2.1 cells maintained eGFP after implantation in immunocompetent, non–eGFP tolerant BALB/c mice. These results are consistent with the recent finding that GFP-specific T cells (just eGFP death inducing, Jedi) are unable to eliminate quiescent subpopulations of mammary tumors despite MHC-I expression (20), and suggest that dormant tumors can be effectively shielded from antigen-specific immunity and selection by other means.

### D2.1 dormant tumor cells have a hybrid E/M phenotype and are enriched in mammary progenitor genes associated with late recurrence in humans.

**Figure 2:**
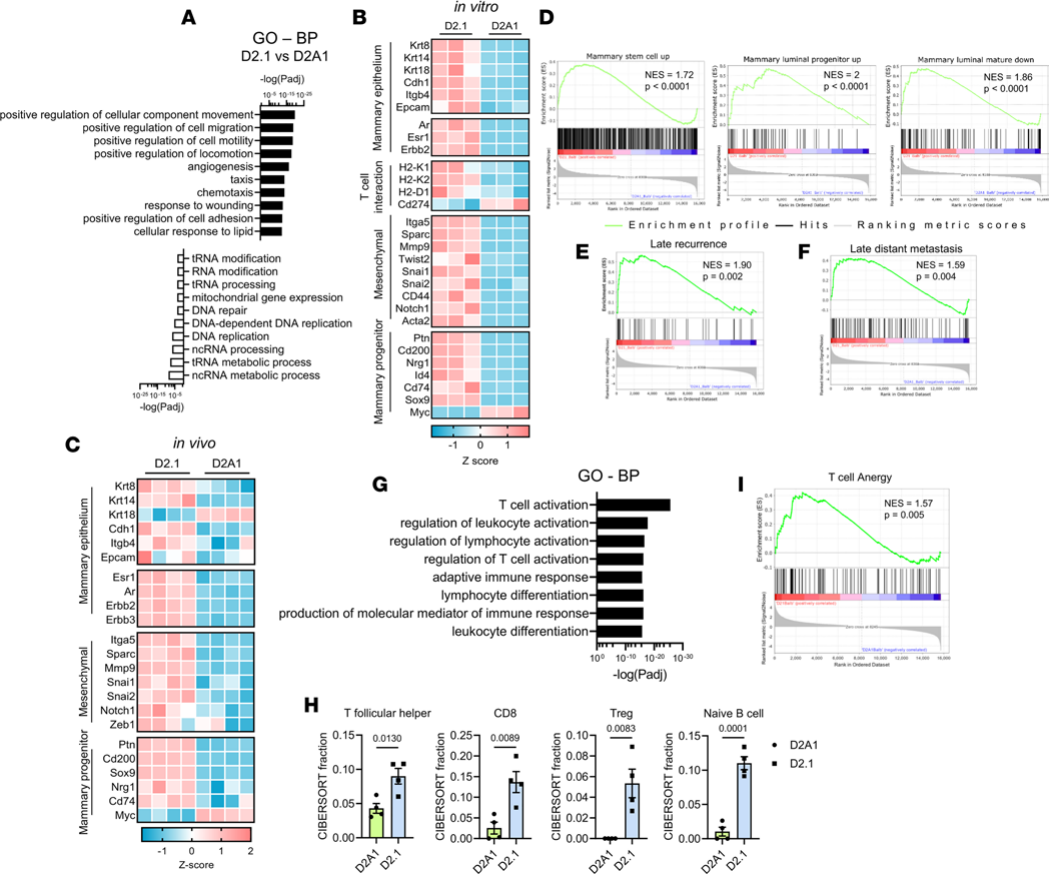
D2.1 tumor cells are enriched in mammary progenitor genes and maintain a hybrid E/M profile in vivo associated with immune dysfunction. (**A**) RNA-seq was performed on D2A1 and D2.1 cells cultured in vitro. Shown are the top 10 upregulated (black fill) and downregulated (white fill) Gene Ontology (GO) Biological Process (BP) pathways in D2.1 compared to D2A1. (**B**) Heatmap of selected significantly differentially expressed genes related to markers of mammary epithelium, antigen presentation via MHC-I, a mesenchymal phenotype, and mammary epithelial cell differentiation is shown. (**C**) D2A1 and D2.1 tumors were implanted into the mammary fad pad (1 × 10^6^/mouse) and collected at approximately 500 mm^3^ for RNA-seq (*n* = 4/group). Shown are selected differentially expressed genes as in **B**. (**D**) GSEA of upregulated gene signatures in D2.1 tumors associated with mammary stem cells, luminal progenitor cells, and luminal mature cells compared to D2A1. (**E** and **F**) GSEA of upregulated gene sets in D2.1 tumors associated with late recurrence (**E**) or late distant metastasis (**F**) in human BC. (**G**) GO BP pathway analysis of D2.1 vs. D2A1 tumors in BALB/c mice. Eight of the top 10 differentially expressed pathways were directly related to immune function. (**H**) CIBERSORT analysis of D2.1 and D2A1 BALB/c tumors. Statistical comparisons were performed by 2-tailed *t* test and data are presented as mean ± SEM. (**I**) GSEA of T cell anergy in D2.1 vs. D2A1 BALB/c tumors.

Due to the striking phenotypic differences in vivo between dormancy-competent D2.1 cells and proliferative counterparts, we initially performed RNA-seq on D2A1 and D2.1 cell lines. The top significantly enriched pathways in D2.1 cells were largely associated with cellular movement and extracellular matrix (ECM) organization, with a significant downregulation of metabolic pathways involved in DNA and RNA processing (Figure 2A and Supplemental Figure 2A). Notably, D2.1 cells expressed higher transcript levels of multiple mammary epithelial maker genes (e.g., *Krt8*, *Krt14*, *Krt18*, *Itgb4*, *Epcam*, and *Cdh1*) and genes formerly associated with mammary progenitor cells (e.g., *Sox9*, *CD74*, *Id4*, *Nrg1*, *Ptn*, and *Cd200*) (Figure 2B). Genes that regulate epithelial-mesenchymal transition (EMT) and metastasis were also upregulated, including genes associated with ECM interaction (*Itga5*, *Sparc*, *Mmp9*, *Cd44*, and *Fn1*) as well as many transcriptional regulators of EMT (*Twist2*, *Snai1*, *Snai2*, and *Notch1*) (Figure 2B). Subsequent quantitative PCR confirmed the hybrid E/M phenotype of D2.1 cells that expressed high levels of epithelial and mesenchymal genes compared with both D2A1 and D2.OR cells (Supplemental Figure 2B). The ERα gene *Esr1* and the ERα target gene *Greb1* were also expressed in D2.1 cells (Supplemental Figure 2C) — an interesting corollary, as human ER^+^ tumors generally display the highest degree of dormancy (5, 21).

In accordance with an overall mammary progenitor–like expression profile, D2.1 cells were found to be CD44^hi^CD24^lo/–^ compared with D2.OR and D2A1 cells by flow cytometry, with D2.OR cells having the most CD24 expression (Supplemental Figure 2D) (22, 23). The presence of increased epithelial and mesenchymal genes could also reflect the described “hybrid epithelial/mesenchymal (E/M)” state associated with surface CD44 and CD104 expression (24, 25). Interestingly, surface expression of CD44 and CD104 revealed largely nonoverlapping populations between D2A1, D2.OR, and D2.1 cells (Supplemental Figure 2E). Because D2.OR tumors are immune sensitive (Figure 1G), overt tumors were often rejected in the MFP of BALB/c animals. However, small, residual nodules that reemerged after approximately 100 days phenotypically resembled D2.1 cells based on CD44/CD104 expression (Supplemental Figure 2F). Altogether, these data indicate that D2.1 cells have a hybrid E/M phenotype at baseline that has been associated with metastasis, dormancy, and immunosuppression (24–27), and may represent a particular subpopulation that can reemerge after immune rejection of the tumor at large (as from D2.OR tumors).

### D2.1 tumor phenotype is maintained throughout progression.

Bulk RNA-seq was also performed on MFP D2A1 and D2.1 tumors from both BALB/c and SCID-beige mice to evaluate phenotypic differences and changes from the selective pressure of adaptive immunity (Supplemental Figure 3A). Overall, D2A1 and D2.1 tumors were largely similar whether in BALB/c or SCID-beige mice (Supplemental Figure 3, B and C). The hybrid E/M and mammary progenitor expression pattern was maintained long-term in D2.1 tumors (Figure 2, C and D) (28). Furthermore, D2.1 tumors were enriched for a genetic profile of late recurrence that we previously developed from human BC samples (29), as well as an independent profile of late distant metastasis also generated from human data (30) (Figure 2, E and F, and Supplemental Table 1). Pathway analysis also revealed that even later-stage D2.1 tumors displayed a less proliferative phenotype when compared with D2A1 tumors in both BALB/c and SCID-beige animals (Supplemental Figure 3, D and E) with upregulated *Cdkn1b* (p27, a common indicator of tumor dormancy; refs. 20, 31) and reduced expression of genes associated with proliferation (Supplemental Figure 3F). Somewhat surprisingly, Gene Ontology (GO) analysis revealed that 8 of the top 10 upregulated pathways in D2.1 tumors specifically related to immune activation (Figure 2G), and while general markers of T cells (*Cd3d*, *Cd3e*, and *Cd3g*) and cytotoxic T cells (*Cd8a* and *Cd8b1*) were associated with tumors from all BALB/c animals, Treg genes (*Ctla4* and *Foxp3*) were only associated with D2.1 tumors in immunocompetent animals (Supplemental Figure 3G). Subsequent CIBERSORT analysis indicated increased CD4^+^ T follicular helper cells, CD8^+^ cells, Tregs, and naive B cells in D2.1 compared with D2A1 tumors (Figure 2H), despite the possibility that these immune cells were less functional (Figure 2I).

As the *Myc* oncogene was significantly upregulated in D2A1 tumors (Figure 2, B and C, and Supplemental Figure 4A), we speculated that the dormant phenotype of D2.1 cells could be reversed by Myc-stimulated proliferation. To test this, we expressed a stabilized form of Myc (T58A) (32) in D2.1 cells (Supplemental Figure 4B). While Myc-T58A expression enhanced proliferation in vitro and in SCID-beige mice, it did not translate to enhanced tumor burden in immunocompetent BALB/c mice (Supplemental Figure 4, C and D). This suggests proproliferative signaling in tumors is insufficient to counteract strong immune editing of proliferative cells, and that a period of dormancy followed by growth is potentially a necessary stage for immune escape in these populations.

### Dormant mammary tumors have high levels of infiltrating FoxP3^+^ Treg cells.

**Figure 3:**
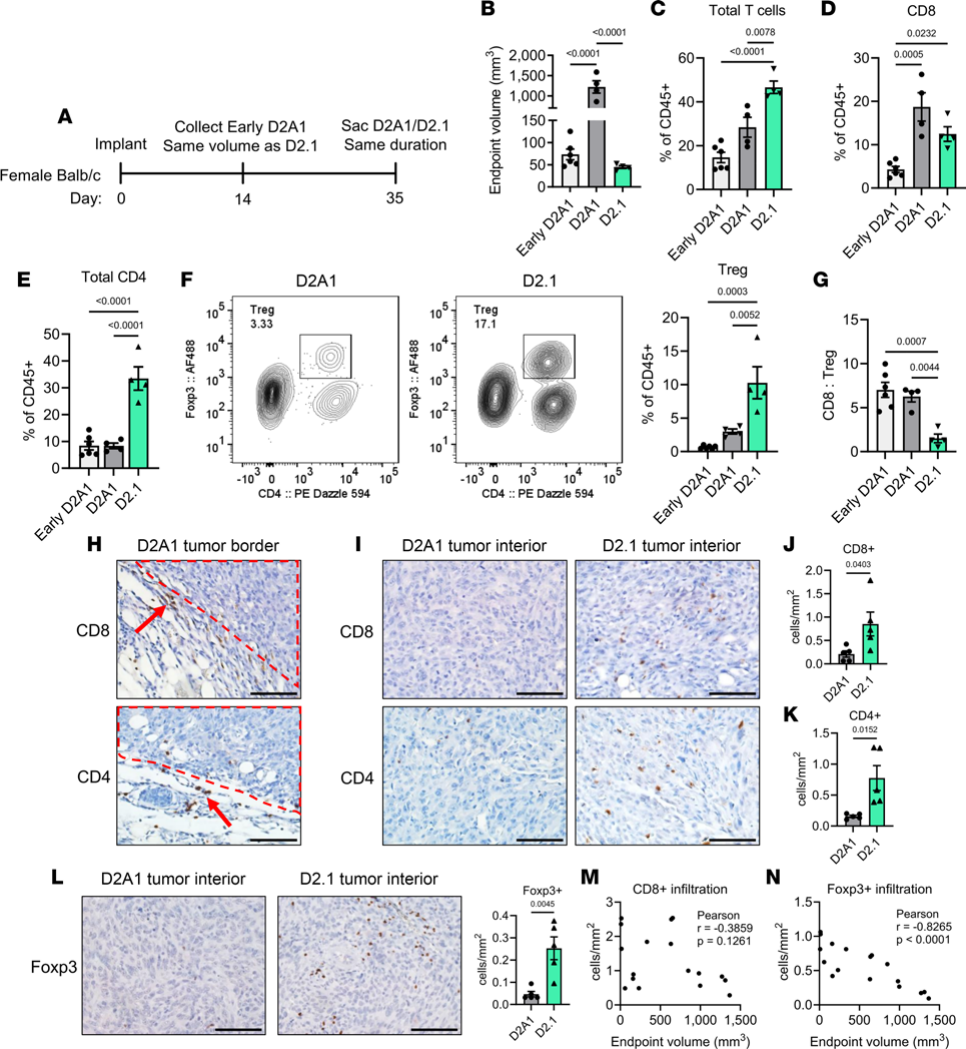
Dormant tumors are highly infiltrated by T cells but induce a Treg-rich microenvironment. (**A**) D2A1 or D2.1 cells were implanted into the mammary fat pad of BALB/c mice (1 × 10^6^/mouse). Small, volume-matched D2A1 tumors (“Early D2A1”; *n* = 6), large, duration-matched D2A1 (“D2A1”; *n* = 4) tumors, or small D2.1 (*n* = 4) tumors were collected as indicated. (**B**) Final volume of tumors in **A**. (**C**) Flow cytometric analysis of total T cells (CD45^+^CD11b^–^CD4^+^ or CD45^+^CD11b^–^CD8β^+^) among total immune cells (CD45^+^) in tumors at time of euthanasia. (**D**) Percentage of CD8^+^ cells among total CD45^+^ cells. (**E**) Percentage of CD4^+^ cells among total CD45^+^ cells. (**F**) Left: Flow plots of FoxP3^+^CD4^+^ cells in D2A1 and D2.1 tumors obtained on day 35 (shown gated on CD45^+^ cells). Right: Quantification of Tregs in tumors from **A**. (**G**) The CD8^+^ cell to Treg (FoxP3^+^CD4^+^) ratio in total T cells by flow cytometry. One-way ANOVA with Tukey’s post hoc analysis was used to compare 3 groups for **B**–**G**. (**H**) Representative IHC image of tumor border from endpoint D2A1 tumors. Dashed lines indicate stromal/tumor interface and arrows indicate positively stained CD8^+^ (top) or CD4^+^ (bottom) cells. (**I**) Representative images of sized-matched D2A1 or D2.1 tumor interiors stained for CD8 (top) or CD4 (bottom). (**J** and **K**) Quantification of CD8^+^ (**J**) or CD4^+^ (**K**) cells/mm^2^ in D2A1 and D2.1 tumor interiors (*n* = 5 each). (**L**) Representative images (left) and quantification (right) of FoxP3^+^ cells in tumors from **H**–**K**. Scale bars: 50 μm (**H**, **I**, and **L**). Density analysis was performed on 5 random fields/tumor using ImageJ software and comparisons were performed via 2-tailed *t* test (**J**–**L**). (**M** and **N**) Correlation of endpoint volume and CD8^+^ density (**M**) or FoxP3^+^ density (**N**) in D2.1 tumors of different sizes (*n* = 17). Pearson’s correlation is displayed in each plot. Data are presented as mean ± SEM.

In light of the immune-independent nature of D2.1 latency, the presence of immune-related genes in D2.1 tumors was striking. Therefore, D2.1 cells were orthotopically implanted and collected after 35 days for immune analysis, and D2A1 tumors were either collected early at the same final size as D2.1 tumors (14 days after implantation) or after the same total duration (35 days after implantation) to ensure that potential differences were not merely due to tumor burden (Figure 3, A and B). Interestingly, flow cytometry revealed that D2.1 tumors indeed contained significantly more total T cells than D2A1 tumors of the same volume or time in vivo (Figure 3C). While large D2A1 tumors had more infiltrating CD8^+^ T cells in comparison with both small D2A1 tumors and D2.1 tumors (Figure 3D), D2.1 tumors harbored more total CD4^+^ cells than D2A1 cells at either stage (Figure 3E). Critically, the Foxp3^+^CD4^+^ Treg population was significantly elevated in D2.1 tumors (Figure 3F), ultimately resulting in a decreased CD8^+^ cell/Treg ratio compared with all D2A1 tumors (Figure 3G).

To ascertain the spatial relationship of T cells with tumor cells and determine whether differences in T cell profiles were maintained through tumor progression, D2.1 tumors were also collected at a later stage (100 days after implantation) (Supplemental Figure 5B). Staining for CD4^+^ and CD8^+^ T cells revealed that both localized at the tumor/stromal interface in size-matched D2A1 tumors (Figure 3H; a feature observed in approximately 26% of TNBCs, ref. 33). However, endpoint D2.1 tumors were highly infiltrated with both CD4^+^ and CD8^+^ T cells (Figure 3I), resulting in a significantly increased interior T cell density in comparison with similarly sized D2A1 tumors (Figure 3, J and K). Furthermore, D2.1 tumors harbored a significant population of infiltrating FoxP3^+^ cells, which likely represents the same FoxP3^+^CD4^+^ Treg population observed via flow cytometry based on multicolor IHC (Figure 3L and Supplemental Figure 5C). No correlation in CD8^+^ cell density was observed collectively across D2.1 tumors of multiple time points and sizes (Figure 3M); however, Foxp3^+^ cell infiltration was significantly correlated with early, smaller tumors (Figure 3N). Thus, dormant tumors do not necessarily restrict T cell infiltration even after beginning to proliferate, indicating that changes in T cell function may be more relevant than proximity. Additionally, these data suggest that Treg induction is an early and necessary event to establish the dormant tumor niche for long-term survival.

### Tumor antigen–specific CD8^+^ cells do not prevent dormant tumor progression in vivo.

**Figure 4:**
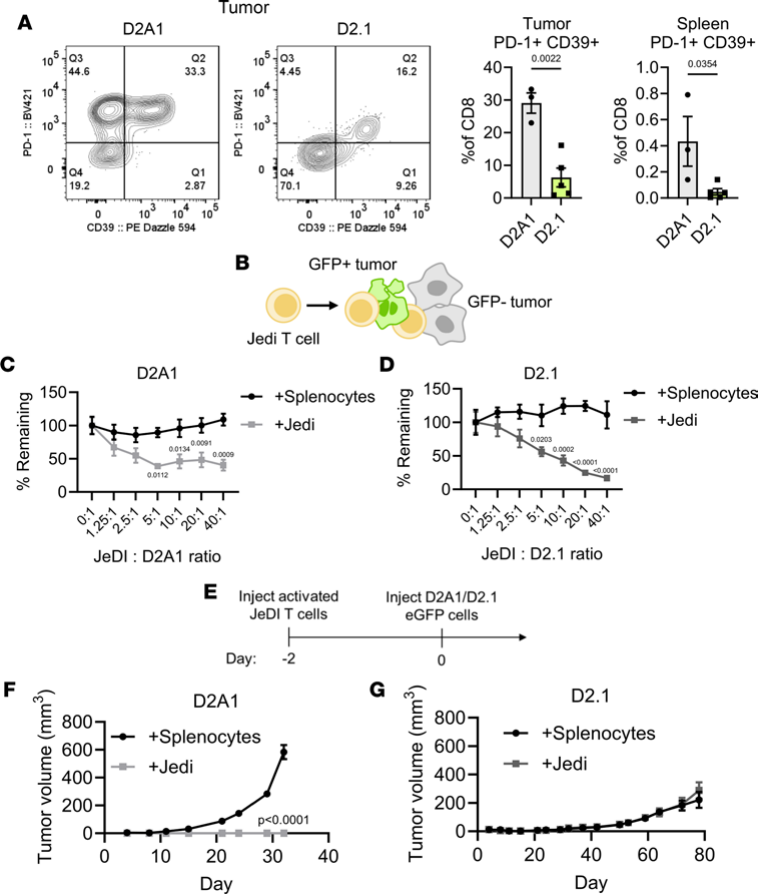
Dormant D2.1 tumors evade antigen-specific killing by activated, tumor-specific CD8^+^ cells. (**A**) Representative flow plots (left) of PD-1 and CD39 expression on CD8^+^ cells from endpoint D2A1 or D2.1 tumors and quantification (right) of PD-1^+^CD39^+^ cells among total CD8^+^ T cells in tumors or spleens of tumor-bearing BALB/c animals. Statistical comparisons were performed by 2-tailed *t* test. (**B**) Jedi T cells express a TCR specific for eGFP_200–208_ that enables antigen-specific targeting of eGFP^+^ tumor cells. (**C**) In vitro killing of D2A1 cells plated with activated Jedi T cells at the indicated ratios. (**D**) In vitro killing of D2.1 cells plated with activated Jedi T cells. Values were normalized to a 0:1 E:T ratio for each condition and were compared by Šídák’s 2-way ANOVA. (**E**) Ex vivo–expanded Jedi T cells or naive splenocytes were transferred to naive mice and 1 × 10^6^ D2A1 or D2.1 eGFP-expressing cells were implanted into the mammary fat pad after 2 days (*n* = 5/group). (**F** and **G**) Growth plots of D2A1 (**F**) or D2.1 tumors (**G**). *P* values calculated by Šídák’s 2-way ANOVA at end of experiment. Data are presented as mean ± SEM.

Primary mammary tumors were recently reported to induce CD8^+^ T cells (CD39^+^PD-1^+^) that systemically control metastatic dormancy in the lungs in the 4T07 model (10). Contrastingly, analysis of MFP D2A1-eGFP and D2.1-eGFP tumors (Supplemental Figure 5E) revealed that D2A1 tumors contained significantly more CD39^+^PD-1^+^CD8^+^ T cells than D2.1 tumors, with a similar trend seen systemically in the spleens of tumor-bearing animals (Figure 4A). Absent a strong, de novo tumor-specific T cell response, we next investigated whether D2.1 persistence could be overcome with potent and systemic antigen-specific T cells. To exclude the role of the tumor microenvironment, killing assays were initially performed in vitro to assess the cytotoxic effect of antigen-specific CD8^+^ T cells against D2.1 and D2A1 cells. Jedi T cells, which express a H-2K^d^–restricted T cell receptor (TCR) for GFP_200–208_ (Figure 4B) (34), were activated/expanded ex vivo and cultured with D2A1 or D2.1 eGFP cells. Both D2A1 and D2.1 cells were equivalently lysed by Jedi cells, thereby verifying that both are susceptible to direct killing by activated Jedi CD8^+^ T cells (Figure 4, C and D). Adoptive transfers were then performed to determine whether externally activated antigen-specific CD8^+^ T cells could prevent/limit eventual D2.1 tumor outgrowth. Activated Jedi cells were injected intravenously into naive C57BL/6 × BALB/c F_1_ mice, and 2 days later D2A1 or D2.1 cells expressing eGFP were implanted into the MFP (Figure 4E). While D2A1 tumor outgrowth was completely prevented by Jedi T cells compared with control T cells (Figure 4F), D2.1 tumors were unaffected by Jedi T cells in comparison to controls (Figure 4G). These results indicate that even highly activated antigen-specific CD8^+^ T cells are incapable of preventing establishment and eventual growth of dormant D2.1 tumors in vivo.

### D2.1 cells secrete factors that induce CD4^+^ and FoxP3^+^ Tregs.

**Figure 5:**
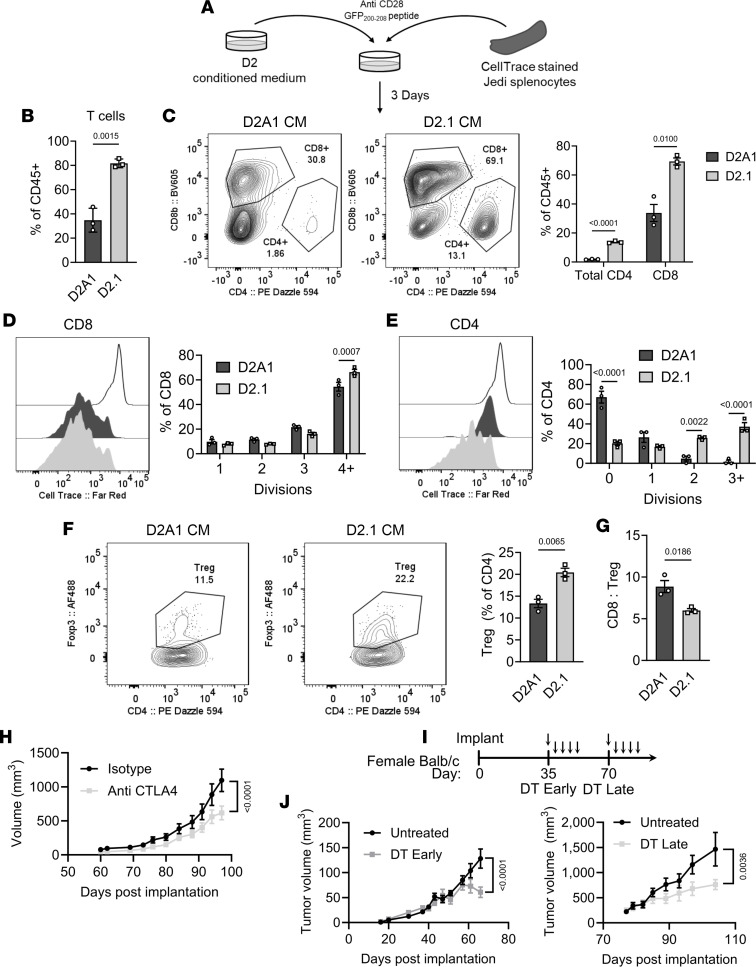
D2.1 cells directly induce CD4^+^ Tregs that can target in vivo. (**A**) Whole Jedi splenocytes were stained with CellTrace and stimulated with anti-CD28 antibodies and GFP_200–208_ peptides in conditioned medium (CM) from D2A1 cells or D2.1 cells for 3 days followed by flow cytometric analysis. (**B**) Total T cells (CD45^+^CD11b^–^CD4^+^ or CD45^+^CD11b^–^CD8β^+^) among immune cells (CD45^+^) at the end of culture. (**C**) Representative flow plots of CD4^+^ and CD8^+^ T cells among CD45^+^ cells and proportion of total CD4^+^ and CD8^+^ cells among CD45^+^ cells. (**D** and **E**) Representative flow plots (left) and quantification of divisions (right) of CellTrace-stained CD8^+^ cells (**D**) or CD4^+^ cells (**E**) in D2A1 or D2.1 CM. (**F**) Representative plots (left) and quantification (right) of FoxP3^+^ Tregs in D2A1 and D2.1 CM after 3 days in culture. (**G**) CD8^+^/Treg ratio in T cells in D2A1 or D2.1 CM. All conditions were performed in triplicate. Statistical comparisons were performed by 2-tailed *t* test (**B**, **C**, **F**, and **G**) or Šídák’s 2-way ANOVA (**D** and **E**). (**H**) Growth of D2.1 tumors (1 × 10^6^ cells/mammary fat pad) treated with anti-CTLA4 antibodies (*n* = 10) or isotype control (*n* = 9) upon reaching 150 mm^3^. (**I**) D2.1 (1 × 10^6^ cells/mammary fat pad) were implanted into female FoxP3-DTR mice and received 0 ng/g DT (Untreated) or 5 doses of 25 ng/g every 4 days (arrows) beginning on day 35 (DT Early) or day 70 (DT late). (**J**) D2.1 tumor volume comparing all Untreated (*n* = 14) mice to DT Early (*n* = 9) at time of DT Early euthanasia (left) or comparing remaining Untreated mice to DT Late (*n* = 8 each) at end of experiment (right). Statistical comparisons for **H** and **J** were performed by Šidák’s 2-way ANOVA and *P* values shown are at the final time point. Data are presented as mean ± SEM.

Increasing evidence suggests that E/M plasticity, as observed in D2.1 cells, is associated with an overall immunosuppressive microenvironment (27). However, because CD8^+^ T cells were distinctly present in but unable to eliminate latent tumors, we investigated whether tumor cells themselves altered T cell function directly. The effect of the tumor secretome on CD8^+^ T cell activation/proliferation was initially to be tested by culturing splenocytes from transgenic Jedi mice with tumor-derived conditioned medium (CM) (35). Whole Jedi splenocytes were stained with CellTrace dye and stimulated with GFP_200–208_ peptides and anti-CD28 antibodies in the presence of D2A1 or D2.1 CM for 3 days (Figure 5A). Overall, D2.1 CM resulted in significantly more T cells than D2A1 CM (Figure 5B), including both CD8^+^ (which was stimulated via peptide) and CD4^+^ cells (Figure 5C). CellTrace staining indicated that CD8^+^ T cells indeed underwent more divisions in D2.1 CM compared with D2A1 CM (Figure 5D); however, CD4^+^ T cells displayed more significantly enhanced proliferation in D2.1 CM in comparison with D2A1 CM (Figure 5E). Further experiments revealed a significant increase in Tregs within the CD4^+^ population (Figure 5F), ultimately resulting in a decreased CD8^+^ cell/Treg ratio in D2.1 CM compared with D2A1 (Figure 5G). Thus, these data suggest that the disparity between D2A1- and D2.1-induced T cell responses in vivo are likely more attributable to dormant tumor cell promotion of CD4^+^ Treg proliferation and/or differentiation via soluble factors.

### Tregs protect D2.1 tumors.

Because CD8^+^ T cell functionality was ostensibly restricted in D2.1 tumors with an associated increase in Tregs, we estimated that Tregs were a more central determinant of immunosuppression in the dormant tumors. Given the ability of anti-CTLA4 IgG2A/B antibodies to deplete intratumoral Tregs (36, 37), we first treated mice bearing D2.1 tumors biweekly with anti-CTLA4 (clone 9D9, 200 μg/mouse) or isotype controls upon the tumors reaching approximately 150 mm^3^. This treatment significantly reduced tumor growth (Figure 5H and Supplemental Figure 6A), with a modest reduction in intratumoral Tregs (although total T cell levels were unchanged) (Supplemental Figure 6B). While these results suggested the importance of Tregs in dormant tumors, anti-CTLA4 antibodies can alter costimulation; thus, we wanted to validate these findings in a more specific Treg model. We therefore implanted D2.1 cells into FoxP3-DTR mice, in which Tregs can be ablated through diphtheria toxin (DT) administration (Supplemental Figure 6C). Tumor-bearing animals were treated with DT every 4 days beginning at an early time point (day 35) or late time point (day 70), with a total of 5 doses each (Figure 5I). Notably, both early and late DT treatment resulted in significant reduction in tumor growth (Figure 5J and Supplemental Figure 6, D and E). Late-DT-treated and control tumors were analyzed upon euthanasia, which showed an increase in infiltrated T cells (Supplemental Figure 6F), including an increase in activated CD39^+^PD-1^+^CD8^+^ T cells. Altogether, these data indicate that despite high CD8^+^ T cell infiltration, the concomitant presence of Tregs in D2.1 tumors restricts antitumor immunity and enables tumor persistence.

### DKK3 is crucial for D2.1 tumor persistence.

**Figure 6:**
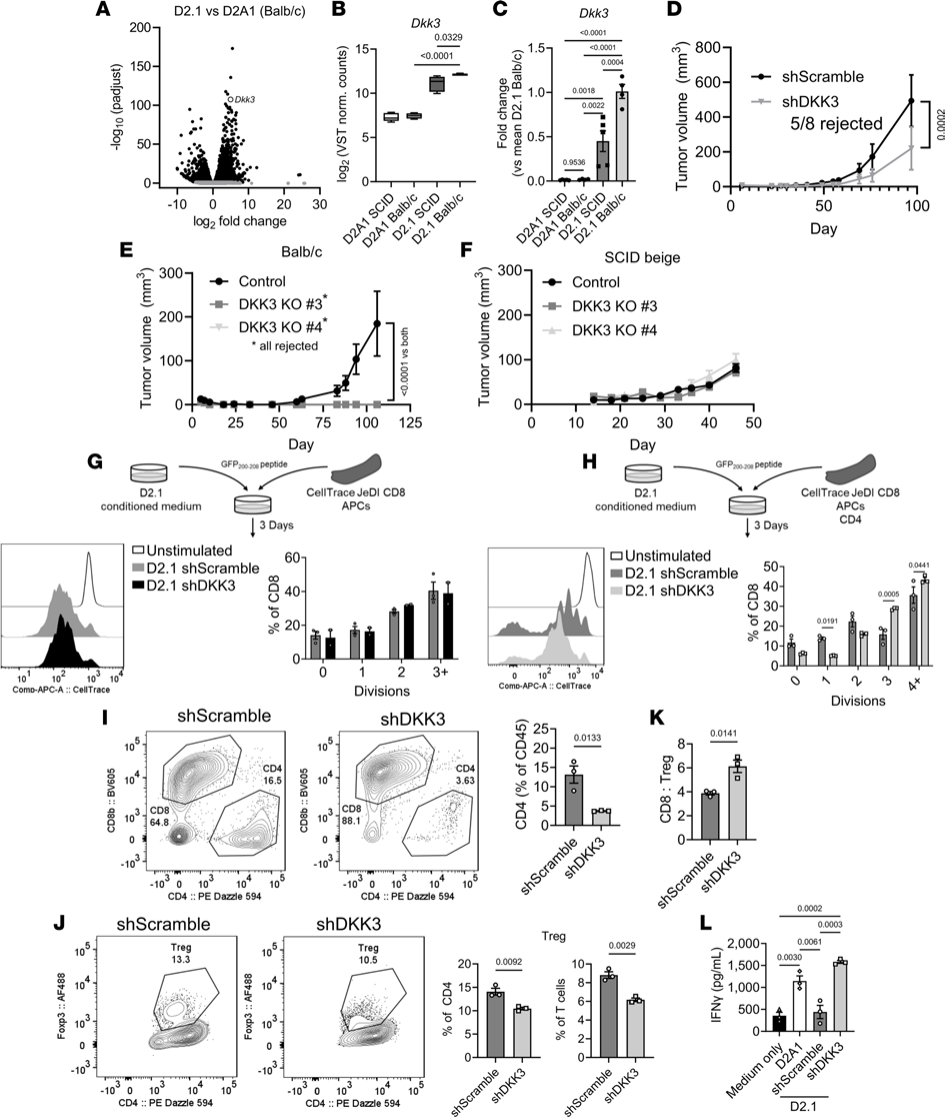
D2.1-derived DKK3 is essential for tumor persistence and mediates T cell fate/function. (**A**) Differential expression analysis of D2.1 vs. D2A1 tumors in BALB/c animals from Figure 2. *Dkk3* is highlighted with an empty circle (log_2_[fold change] = 4.97; *P*_adj_ = 3.14 × 10^–108^). (**B**) Normalized counts of *Dkk3* from bulk RNA-seq of D2.1 or D2A1 tumors implanted into BALB/c or SCID-beige animals. Counts were compared by 1-way ANOVA with Holm-Šídák multiple-comparison test. (**C**) Quantitative PCR of *Dkk3* transcripts in samples from **B**. *P* values by 1-way ANOVA with Holm-Šídák multiple-comparison test. (**D**) Growth of 1 × 10^6^ D2.1 cells expressing shScramble (*n* = 6) or shDKK3 (*n* = 8) vectors in the mammary fat pad (MFP) of BALB/c mice. (**E**) Growth of 1 × 10^6^ D2.1 control (*n* = 10) or 2 independent DKK3-KO lines (*n* = 5 each) in the MFP of BALB/c mice. Statistical comparisons were by 2-way ANOVA at end of experiment (**D** and **E**). (**F**) Growth of D2.1 control or DKK3-KO cells (*n* = 5 each) in the MFP of SCID-beige mice. (**G**) Bead-isolated Jedi CD8^+^ cells were cultured in D2.1 shScramble or shDKK3 CM with antigen-presenting cells (APCs) and eGFP_200–208_ peptide. Representative flow plot (left) and quantification (right) of CD8^+^ cell divisions in D2.1 CM are shown. (**H**) Jedi CD8^+^ cells cultured as in **G** with the addition of equal numbers of bead-isolated CD4^+^ cells. (**I**) Representative plots of CD4^+^ and CD8^+^ cells (gated on CD45^+^; left) and quantification (right) of CD4^+^ cells among CD45^+^ cells in D2.1 CM. (**J**) Representative plots (left) and quantification of Tregs in D2.1 CM. (**K**) CD8^+^ cell/Treg ratio after culture in D2.1 CM. (**L**) IFN-γ ELISA after T cell culture in D2.1 CM. Statistical comparisons were performed by 2-tailed *t* test (**I**–**K**), 1-way ANOVA with Tukey’s correction (**L**), or Šídák’s 2-way ANOVA (**G** and **H**). Data are presented as mean ± SEM.

Knowing that tumor-derived secreted factors were sufficient to regulate the T cell landscape we further investigated potential soluble mediators of this effect. The hybrid E/M and mammary progenitor-like signature of D2.1 tumors was typified by high expression of DKK3, a Wnt signaling modulator that is preferentially expressed by CD44^+^CD24^–^ epithelial stem/progenitor cells within the human mammary gland and is generally enriched across stem cell or “immune privileged” niches, making it an attractive candidate (Figure 6A) (38, 39). Interestingly, DKK3 expression was even greater in D2.1 tumors in immunocompetent animals but not D2A1 (Figure 6, B and C). Elevated DKK3 expression in D2.1 cells compared with D2A1 or D2.OR cells was confirmed via quantitative PCR (Supplemental Figure 7A) and its function was assessed after stable shRNA knockdown in D2.1 cells (Supplemental Figure 7, B and C). Upon MFP implantation, shDKK3 D2.1 tumors displayed significantly reduced growth in vivo compared with shScramble controls, with the majority being completely rejected (5/8 mice; Figure 6D and Supplemental Figure 7D). Subsequent analysis of tumor cells after expansion ex vivo confirmed that nonrejected shDKK3 tumors regained DKK3 expression — likely explaining the persistence of these tumors (Supplemental Figure 7E). To validate these results, 2 independent CRISPR DKK3-knockout (DKK3-KO) D2.1 clones were generated (Supplemental Figure 7, F and G). When implanted into immunocompetent animals DKK3-KO cells were completely rejected (Figure 6E), whereas in SCID-beige animals DKK3-KO cells yielded tumors equivalent to control cells (Figure 6F). Thus, DKK3 expression indeed appears to be a necessary component for immune escape and long-term survival during the dormant phase.

### Tumor-derived DKK3 regulates Tregs to inhibit CD8^+^ T cell function.

The mechanistic underpinnings of DKK3-mediated immune evasion were further validated ex vivo by stimulating isolated CD8^+^ Jedi T cells mixed with antigen-presenting cells (APCs) in D2.1 shScramble or shDKK3 CM. Unexpectedly, no difference in CD8^+^ T cell proliferation was detected between D2.1 shDKK3 CM and control (Figure 6G). However, additional experiments using a mix of CD8^+^ Jedi cells, CD4^+^ cells, and APCs revealed significantly increased total T cells in shDKK3 CM compared with shScramble (Figure 6H), with increased CD8^+^ T cell proliferation, suggesting an indirect effect of DKK3 on CD8^+^ T cells. Consistent with this observation, the CD4^+^ T cell fraction was significantly reduced in DKK3-silenced D2.1 CM (Figure 6I), with a decrease in FoxP3^+^CD4^+^ Tregs (Figure 6J), yielding an increased CD8^+^ cell/Treg ratio (Figure 6K). Similar results were also observed when using CM from DKK3-KO cells (Supplemental Figure 8, A–C). As a proxy for immune activation, we also assessed the level of IFN-γ in media by ELISA and found that DKK3 knockdown resulted in increased IFN-γ compared with control D2.1 CM and was comparable to culturing in D2A1 CM (Figure 6L). Altogether, these experiments support the previous data and implicate CD4^+^ T cells, particularly Tregs, as a target of D2.1 regulation via secretion of DKK3, with secondary effects on CD8^+^ cells.

### DKK3 is associated with poor survival and immune evasion in human BC.

**Figure 7:**
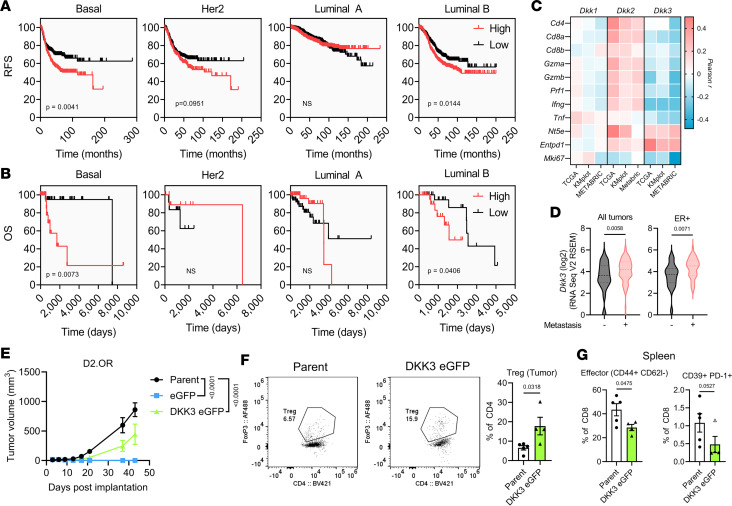
DKK3 is associated with poor survival, decreased effector function, and metastasis in human BC and promotes tumor growth in an immunocompetent setting. (**A**) Survival plots of human BC stratified by high and low *Dkk3* expression in the Kaplan-Meier Plotter cohort (Basal = 477, Her2 = 348, Luminal A = 903, Luminal B = 676). (**B**) Survival plots of TCGA human BCs based on *Dkk3* expression. (**C**) Correlation analysis of *Dkk1*, *Dkk2*, and *Dkk3* with immune-related genes in TCGA, Kaplan-Meier Plotter, and METABRIC data sets. Individual *P* values are provided in Supplemental Table 2. (**D**) *Dkk3* expression in MBCProject samples with or without metastases present at time of sample collection. Statistical comparisons were performed by 2-tailed *t* test. (**E**) Growth of parental, eGFP-expressing only, or DKK3 eGFP D2.OR mammary fat pad tumors (1 × 10^6^ cells; *n* = 5 each) in female BALB/c mice. Comparisons shown are by Tukey’s 2-way ANOVA at end of experiment. (**F**) Representative flow plots (left) and quantification (right) of Tregs in total CD4^+^ cells in tumors from **E**. (**G**) Effector phenotype (CD44^+^CD62L^–^) of CD8^+^ T cells in spleens of mice from **E**. Statistical analysis of **F** and **G** was performed by 2-tailed *t* test. An outlier was identified for **G** (right; empty triangle) using the outlier analysis function in GraphPad Prism (ROUT method, *q* = 1%) and the *P* value displayed excludes the outlier (*P* = 0.1309 included). Data are presented as mean ± SEM.

Given the experimental evidence of DKK3-modulated immune function, DKK3 was assessed in BC data sets obtained from clinical samples to determine the relevance of this pathway in humans. In BC, we found that high *Dkk3* expression was significantly correlated with poor survival in Basal and Luminal B tumors in both the Kaplan-Meier Plotter and TCGA data sets (Figure 7, A and B). Interestingly, *Dkk3* was inversely correlated with genes indicative of an antitumor immune response (e.g., *Cd4*, *Cd8a*, *Gzma*, *Gzmb*, and *Ifng*) in Basal BC across data sets, a pattern not observed with family member *Dkk1* or *Dkk2* (Figure 7C and Supplemental Table 2). Furthermore, *Dkk3* was positively correlated with the ectonucleotidases *Nt5e* (CD73) and *Entpd1* (CD39), which are highly expressed by Tregs and critically mediate peripheral tolerance (40), and negatively correlated with the proliferation marker *Mki67* (Figure 7C). Expression of *Dkk3* was also higher in primary tumors of BC patients who presented with metastasis, even in ER^+^ tumors that have higher *Dkk3* at baseline (Figure 7D and Supplemental Figure 9A). Single-cell RNA-seq analysis of human BCs revealed that *Dkk3* is expressed by multiple cells within the breast microenvironment, including both normal and cancer epithelial cells such as TNBC cells (Supplemental Figure 9, B and C). Thus, in human BC both the stroma and tumor cells themselves likely contribute DKK3 to the microenvironment (41).

### DKK3 promotes immune evasion in rapidly progressing models of BC.

To directly assess the role of DKK3 in suppressing antitumor immunity, we utilized lentiviral vectors to stably overexpress DKK3 in D2.OR cells, which we found to be highly sensitive to adaptive immune responses (Supplemental Figure 10A and Figure 1G). While DKK3 expression yielded no significant difference in cellular proliferation in vitro, it did elicit a CD44/CD104 profile similar to D2.1 cells or residual D2.OR tumors (Supplemental Figure 10, B and C). Upon MFP implantation in immunocompetent hosts, DKK3 expression provided a robust engraftment and survival advantage compared with control empty vector D2.OR tumors, which were rejected after stable integration of the puromycin xenoantigen (Supplemental Figure 10D). Prior reports suggest that DKK3 enables MHC-I–mismatched transplantation of embryoid bodies (42); therefore, to test the possibility that DKK3 protected against foreign antigen–specific immunity, a vector containing eGFP in addition to DKK3 was lentivirally transduced into D2.OR cells, which were subsequently transplanted into the MFP of BALB/c mice. As with puromycin-expressing D2.OR cells, we found that D2.OR cells expressing eGFP alone were completely rejected, while parental D2.OR cells formed tumors (Figure 7E). However, D2.OR cells expressing both eGFP and DKK3 successfully engrafted, although at a diminished rate compared with parental cells (Figure 7E). Moreover, examination of infiltrating T cells revealed an increase in Tregs compared with parental tumors (Figure 7F) and a systemic decrease in effector CD8^+^ cells in the spleen (Figure 7G). Together, these data suggest that DKK3 enables antigen-specific protection in otherwise immune-sensitive tumors and supports a role for DKK3 in Treg generation.

Congruent results were observed when expressing DKK3 in highly proliferative D2A1 cells (Supplemental Figure 10F). We found that DKK3-expressing D2A1 cells grew equivalently whether implanted into the MFP of BALB/c or SCID-beige mice, whereas control tumors were substantially delayed in BALB/c animals (Supplemental Figure 10G), and ELISA of serum at time of euthanasia confirmed that DKK3 expression was maintained (Supplemental Figure 10H). By the end of the experiment, effector CD8^+^ T cells were significantly reduced in the tumor (Supplemental Figure 10I) and large changes in splenic T cells were detected (Supplemental Figure 10J), with T cells that were shifted toward a naive (CD44^–^CD62L^+^) phenotype. Fewer anti-eGFP antibodies were also present in the serum of mice bearing DKK3-expressing D2A1 tumors when accounting for tumor volume (Supplemental Figure 10K). In sum, these in vivo data reveal that DKK3 can restrict adaptive antitumor immunity during multiple stages of tumor progression.

## Discussion

Tumor dormancy and reemergence depend on both tumor-intrinsic and -extrinsic mechanisms that are slowly being uncovered. Our study does not address how tumors enter and exit dormancy (although others have recently shed light on dormancy in D2 cells, ref. 7); however, we describe an avenue by which intrinsically dormant and tumor populations evade adaptive immunity to eventually form progressive disease. This is achieved via early initiation of a CD4^+^/Treg–skewed T cell response, and we provide evidence that the tumor-derived Wnt modulator DKK3 is critical to this process. We also demonstrate that DKK3 is essential for tumor survival during the latent phase and generally protects tumors from antigen-specific CD8^+^ T cell immunity. Finally, our analysis suggests that DKK3 is a relevant target in BC, particularly in TNBC and Luminal B tumors.

An important determinant of recurrence is the duration that CD8^+^ T cells maintain in their cytotoxic dominance against proliferative tumor cells (43, 44). Evidence suggests that CD39^+^PD-1^+^CD8^+^ T cells represent a locally induced, tumor-reactive population that can also prevent metastatic outgrowth in BC and others (10, 45). However, our studies revealed that dormant tumor cells prevent generation of this CD8^+^ T cell population compared with more proliferative tumors. Critically, we demonstrate that activated tumor antigen-specific CD8^+^ T cells were unable to eliminate these latent tumors, but that targeting Tregs specifically allowed the generation of more effective antitumor responses, suggesting a primacy of Tregs in protecting dormant cancer cells. The degree to which dormant tumors are targetable by CD8^+^ T cells in general also remains elusive. It is commonly understood that MHC-I downregulation in dormant/disseminated tumor cells can promote immune escape (17, 46, 47), although our data suggest the importance of microenvironmental avenues of immune evasion as opposed to being “invisible” to adaptive immunity. A growing body of literature indicates that MHC-I/antigen presentation is highly dynamic (43, 48), supportive of other studies that have found no differences in antigen presentation pathway genes or MHC-I expression in dormant BC cells (20). Collectively, these studies suggest that, although they are in fact detectable by CD8^+^ T cells, many dormant BC tumor cells rely on other means to escape elimination.

While Tregs are generally important for tumor immune evasion (49), a definitive role in protecting dormant tumor cells remains elusive (50). Clinical studies suggest that Tregs are specifically associated with late relapse (>5 years) and poor metastatic survival in BC (51, 52). We found direct evidence that tumor-derived DKK3 was able to induce Treg differentiation in vitro, which ultimately resulted in decreased CD8^+^ T cell function. While others have reported direct effects of DKK3 on CD8^+^ cells (42, 53, 54), we found the effect was most prominent on CD4^+^ T cells/Tregs. Recently, DKK3 was described to regulate downstream Wnt/β-catenin genes *Tcf7,* which encodes T cell factor-1 (TCF-1), and *Lef1* (encoding lymphoid enhancer binding factor 1), in CD4^+^ cells to coordinate IFN-γ secretion by a yet to be determined receptor (55). Canonical Wnt signaling also inhibits Treg activity (56); therefore, suppression of Wnt by tumor-derived DKK3 may mediate CD4^+^ cell differentiation toward Tregs. Additionally, other studies support a role for DKK3 in concert with other factors in the extracellular milieu, such as TGF-β (57–60). The TGF-β pathway is especially relevant for Treg function (61), and because D2.1 cells also expressed more *Tgfb1* we speculate that this may be an important connection to delineate the contextual function of DKK3 on T cells in addition to Wnt.

Currently, the role of DKK3 in cancer is poorly understood, with both inhibitory and beneficial functions being reported in tumors (41, 62–68). In our hands, DKK3 did not directly affect tumor cell proliferation, but instead showed tumor-promoting effects when in contact with the immune system. This is consistent with recent studies in pancreatic tumors that demonstrated increased T cell accumulation in DKK3-KO mice and others showing greater tumor rejection with enhanced CD8^+^ T cell infiltration into tumors when mesenchymal stem cells were DKK3 deficient (54, 69). More generally, DKK3 was identified as necessary for generating antigen-specific, tolerant CD8^+^ T cells in vivo and could suppress T cell proliferation in vitro (53). In an autoimmune context, DKK3 produced locally by stromal cells in the skin was found to reduce self-antigen–induced experimental autoimmune encephalomyelitis symptoms in a CD4^+^ T cell–dependent fashion (42, 70). As such, our results further elucidate an ability of DKK3 to restrain adaptive immunity in normal and pathological conditions through the induction of Tregs, although the exact molecular drivers of this process will need further clarification.

Along with other secreted factors, other cell types in specific niches may play a critical role in DKK3 expression and function. Our results were obtained in an orthotopic site (i.e., the MFP) with tumor-derived cytokines. As mentioned previously, within the human mammary gland *Dkk3* is one of the most upregulated genes in CD44^+^CD24^–^ (stem) versus CD44^–^CD24^+^ (differentiated) cells (38), which mimics the phenotype of D2.1 versus D2A1/D2.OR cells. However, other cells within the local tumor microenvironment and certain disseminated niches may also be important contributors of DKK3. Interestingly, DKK3 is commonly expressed by stromal cells in “immune privileged” and stem cell niches in the brain, eye, pancreas, liver, and bone marrow (39, 71–76). The trophectoderm, which induces immune tolerance at the maternal-fetal interface, in part by recruiting and/or inducing Tregs, is also enriched for DKK3 expression (77–79). Thus, DKK3 may be a component of larger mechanisms that maintain stem cell niches and prevent aberrant immune activation against these critical populations, which we speculate is driven by the plastic E/M phenotype (12), and the fact that DKK3 can prevent MHC-I–mismatched embryonic body rejection (42) supports this notion. Currently, immune checkpoint blockade (ICB) remains largely unsuccessful in hormone receptor–positive BCs (which are most likely to go dormant) due to low T cell infiltration and an immunosuppressive stroma (80). Although pancreatic cancer is similarly resistant to ICB, combined anti-CTLA4 and anti-DKK3 antibodies showed synergistic antitumor responses in a pancreatic cancer model (54). Although ICB has been approved for certain TNBCs, great interest also remains in combinatorial therapies that target alternative pathways to boost ICB response (81). Therefore, targeting DKK3 alongside other immunotherapeutic strategies may help break alternative suppressive barriers to eliminate dormant, residual tumor cells and more aggressive cells alike.

## Methods

### Mice

SCID-beige (C.B-*Igh*-1b/GbmsTac-*Prkdc^scid^*-*Lyst^bg^* N7; model CBSCBG) and BALB/c (BALB/cAnNTac; model BALB) were purchased from Taconic Biosciences. Jedi mice (*Ptprc^a^ Tcrb^Ln1Bdb^ Tcra^Ln1Bdb^ H2d*/J; strain 028062) were purchased from The Jackson Laboratory and subsequently crossed with *Rag1^–/–^* (B6.129S7-*Rag1^tm1Mom^*/J; strain 002216) from The Jackson Laboratory. FoxP3-DTR mice (B6.129(Cg)-*Foxp3^tm3(DTR/GFP)Ayr^*/J; strain 016958) were purchased from The Jackson Laboratory and female FoxP3-DTR mice were crossed with male BALB/c to generate F_1_ animals for implantation. Genotyping and genetic monitoring for all strains were routinely performed by Transnetyx. Female mice between 8 and 12 weeks of age were used for tumor studies.

### Cell lines

D2A1, D2.OR, and D2.1 cells were provided by Ann Chambers (London Health Sciences Centre, London, Ontario, Canada). Cell lines were confirmed mycoplasma negative via MycoStrip (InvivoGen) and were maintained in low-glucose DMEM (Gibco) containing 10% FBS and penicillin (10 U/mL)/streptomycin (10 μg/mL) at 37°C and 5% CO_2_. For a summary of D2 cell phenotypes see Supplemental Table 3.

### Plasmids

The LeGO-G lentiviral plasmid (Addgene, 27347) was used to generate eGFP^+^ D2A1, D2.OR, and D2.1 cells. shRNA lentiviral vectors (pLKO.1) targeting *Dkk3* were obtained from Sigma-Aldrich (TRCN0000071752; target sequence TCTGTGACAACCAGAGGGATT). Gene targeting of *Dkk3* by CRISPR/Cas9 was accomplished via pLentiCRISPRv2 (Addgene, 52961) and sgRNA sequences for *Dkk3* were obtained from the GeCKO v2 CRISPR library provided by Feng Zhang (Addgene, 1000000052) (82). Inducible *Dkk3* vectors were modified from previously published vectors (83). Cloning details are available upon request.

### Lentiviral production and infection

All lentiviral vectors were produced in 293T cells using second-generation packaging plasmids and previously described techniques and viral stocks were concentrated by ultracentrifugation. Viral stocks were added to medium containing 8 μg/mL polybrene and cells were incubated for 48 hours before subculturing. Transduced cells were selected via puromycin (shRNA knockdown, CRISPR knockout) or sorting for eGFP^+^ cells. Single-cell clones were generated by limited dilution in 96-well plates and subsequent culture of wells containing a single colony.

### Orthotopic implantation

Tumor cells in sterile PBS were implanted into the fourth inguinal MFPs under general anesthesia. Tumors were monitored by caliper and volume was calculated using the formula, volume = (length × width^2^) ÷ 2.

### Antibody/DT treatment

Antibodies against CTLA4 (BioXCell, clone 9D9) were administered intraperitoneally (i.p.) biweekly at 200 μg/mouse. DT (Cayman Chemical) was given i.p. at 25 ng/g every 4 days, with a total of 5 doses.

### T cell activation and adoptive transfer

Six-well plates were precoated overnight with 0.5 μg/mL anti-CD3ε (BioXCell, clone 145-2C11) and 5 μg/mL anti-CD28 (BioXCell, clone 37.51) in PBS (5 mL/well) at 4°C. Jedi spleens were crushed through a 70-μm filter, red blood cells were lysed with ACK buffer, and cells were plated at 5 × 10^6^ cells/mL and 5 mL/well in RPMI 1640 medium containing 10% FBS, L-glutamine (2 mM), penicillin (10 U/mL)/streptomycin (10 μg/mL), 2-mercaptoethanol (50 μM), and 1× Insulin-Transferrin-Selenium (ITS -G; Gibco) and cultured overnight at 37°C and 5% CO_2_. The following day, recombinant mouse IL-2 (30 U/mL; BioLegend) and IL-7 (0.5 ng/mL; BioLegend) were added to the culture medium and cells were incubated at 37°C for 24 hours. Cells were subsequently subcultured at 1 × 10^6^ cells/mL in fresh medium containing IL-2 and IL-7 and incubated an additional 24 hours. Expanded T cells were washed twice, resuspended in sterile PBS at 1 × 10^6^ cells/50 μL, and injected i.v. into mice under general anesthesia (1 × 10^6^ cells/mouse).

### Killing assays

D2A1 or D2.1 target cells expressing eGFP-Luc were plated at 2,000 cells/well in a 96-well plate and allowed to attach overnight at 37°C. The following day, serial dilutions of effector Jedi T cells (activated as for adoptive transfer) were added to each well in quadruplicate and plates were incubated overnight at 37°C. Cells were lysed with a Triton X-100–containing buffer and luciferase activity was measured using a Veritas microplate luminometer (Turner Biosystems). The fraction of remaining cells in each well was normalized to the average signal of control wells that did not receive Jedi cells.

### CM harvest

Tumor cells were seeded in complete medium at 5 × 10^3^ cells/mm^2^ in a 10-cm dish and allowed to attach before changing medium to low-glucose DMEM containing 1% FBS. Cells were cultured for 48 hours, at which point CM was collected, centrifuged to remove debris, passed through a 0.45-μm filter, and stored at 4°C until use.

### T cell activation/proliferation assays

#### CD4^+^/CD8^+^ T cell isolation.

Splenocytes were resuspended in EasySep buffer (STEMCELL Technologies) at 1 × 10^6^ cells/mL and CD4^+^ cells were isolated with an EasySep Mouse CD4^+^ T Cell Isolation Kit (STEMCELL Technologies) or CD8^+^ cells were isolated using the EasySep Mouse CD8^+^ T Cell Isolation Kit (STEMCELL Technologies) according to the manufacturer’s protocol.

#### CellTrace staining.

Single cells (whole splenocytes or CD8^+^ cells only) were resuspended in PBS at 1 × 10^6^ cells/mL. Cells were stained with 1 μL/mL (after reconstitution in 20 μL DMSO) CellTrace Far Red (Invitrogen) for 20 minutes at 37°C with gentle mixing every 5 minutes. The cell suspension was diluted 5-fold with complete medium and incubated at room temperature for 5 minutes and washed once with medium.

#### CM culture.

For experiments with whole splenocytes, CellTrace-stained Jedi splenocytes were plated in a 1:1 mixture of D2 CM/fresh medium containing anti-CD28 antibodies (2 μg/mL), eGFP_200–208_ peptide (1 μg/mL), and 2-mercaptoethanol (50 μM) at 7.2 × 10^5^ cells/well in a 24-well plate. Cells were cultured at 37°C for 72 hours and floating/weakly attached cells were stained for Live/Dead, CD45, CD4, CD8β, and FoxP3. For experiments with CD8^+^ cells alone, 4.1 × 10^5^ CellTrace-stained Jedi *Rag1^–/–^* CD8^+^ cells/well and 4.1 × 10^5^ splenocytes from SCID-beige mice (serving as APCs expressing H-2K^d^) were plated with eGFP peptides as before. For experiments with CD8^+^ cells, CD4^+^ cells, and APCs, 2.4 × 10^5^ CD4^+^ cells (from BALB/c), 2.4 × 10^5^ CellTrace-stained Jedi *Rag1^–/–^* CD8^+^ cells, and 2.4 × 10^5^ SCID-beige splenocytes were plated per well with eGFP peptides as before.

### IHC/IF

Formalin-fixed, paraffin-embedded tissue sections were deparaffinized with xylene and rehydrated through graded concentrations of ethanol and distilled water. Epitope retrieval was performed in a Retriever 2100 (Aptum Biologics) with R-Buffer A (Electron Microscopy Sciences).

#### IHC.

Endogenous peroxidases/phosphatases were quenched with BLOXALL blocking solution (Vector) and tissues were blocked with Animal-Free Blocker R.T.U. (Vector). Sections were probed with primary antibodies (for a complete list see Supplemental Table 4) overnight at 4°C, washed with PBS, and incubated with the appropriate ImmPRESS polymer detection reagent (Vector) for 30 minutes at room temperature. Visualization was performed by incubation with 3,3′-diaminobenzidine (DAB) (Vector), ImmPACT Vector Red (Vector), or a Green HRP staining kit (Novus), For triple IHC, a second round of retrieval was performed with R-Buffer A after developing the first round of HRP and AP stains. Tissues were counterstained with Gill No.3 Hematoxylin (Sigma-Aldrich), coverslipped, and imaged on an Olympus IX73 inverted microscope with a 40× objective. Infiltrated T cells were enumerated on 5 random fields of view per sample using ImageJ software (NIH).

#### IF.

Tissues were blocked with Animal-Free Blocker R.T.U. and incubated in primary antibodies overnight at 4°C. Tissues were washed with PBS, and secondary staining was performed for 1 hour in the dark at room temperature with the appropriate fluorophore-conjugated antibody. Tissues were counterstained with DAPI and coverslipped with VECTASHIELD Vibrance (Vector) mounting media. Whole-slide images were acquired using a Zeiss LSM 880 confocal microscope. Ki67 staining analysis was performed using CellProfiler software (https://cellprofiler.org/). Single nuclei were segmented using the DAPI channel and the eGFP channel was used to create a binary mask to delineate between tumor and stroma. The number of Ki67^+^ nuclei within the eGFP mask was quantified and is represented as a percentage of the total nuclei within the eGFP mask.

### Tumor cell proliferation assays

Cells were plated at 4 × 10^3^ cells/well in a 96-well plate and cultured at 37°C. Cells were incubated in medium containing 0.5 mg/mL 3-[4,5-dimethylthiazol-2-yl]-2,5-diphenyltetrazolium bromide (MTT) for 4 hours at 37°C, gently washed, and crystalline formazan was dissolved in DMSO. Plates were read at 550 nm with a Bio-Rad 680 microplate reader.

### Quantitative real-time PCR

RNA was isolated using Qiagen RNeasy kits and reverse transcribed using an iScript cDNA Synthesis Kit (Bio-Rad) before performing quantitative PCR using Sso Advanced Universal SYBR Green Supermix (Bio-Rad). Values were determined using the ΔΔCT method with actin as internal control. Primer pairs can be found in the supplemental material.

### Flow cytometry

Tumors were digested in serum-free medium using collagenase (1 mg/mL), DNase (20 U/mL), and hyaluronidase (100 μg/mL) for 90 minutes at 37°C. Spleens were mechanically dissociated as before. Samples were washed with serum-containing medium, red blood cells were lysed with ACK buffer, and passed through a 40-μm cell strainer to generate single-cell suspensions. For each condition, 2 × 10^6^ cells were stained at 4°C with Fc Block (CD16/32 clone 93; BioLegend), Live/Dead Fixable Dye (Invitrogen), and a combination of directly conjugated antibodies (for a complete list see Supplemental Table 4). Cells were fixed in 1% paraformaldehyde and analyzed on a BD LSR or Cytek Northern Lights flow cytometer. Compensation was performed using single-color-stained splenocytes or BD CompBeads in FlowJo software (v10), which was also used for downstream analysis. After live gating, T cells were identified as CD45^+^CD11b^–^CD4^+^/CD8^+^, CD4^+^ cells were identified as CD45^+^CD11b^–^CD4^+^ and divided into Tregs (CD45^+^CD11b^–^CD4^+^CD8^–^FoxP3^+^) or T helper (CD45^+^CD11b^–^CD4^+^CD8^–^FoxP3^–^), and CD8^+^ cells were identified as CD45^+^CD11b^–^CD8^+^ (Supplemental Figure 5A).

### Quantitative ELISA

Mouse IFN-γ in T cell cultures was detected using the ELISA MAX Set from BioLegend according to the manufacturer’s protocol. Anti-mouse DKK3 ELISA kits were purchased from RayBiotech and serum DKK3 was detected according to the standard protocol.

### RNA-seq

Individual tumors were snap frozen and stored in RNAlater-ICE (Invitrogen) until RNA extraction. Total RNA was extracted using PureLink RNA mini Kits (Invitrogen) and DNA was removed via DNA-free DNA Removal kit (Invitrogen). RNA quality and concentration were determined using an Agilent 2100 Bioanalyzer. Whole transcriptome sequencing of cell lines and tumors was performed by Novogene on an Illumina NovaSeq 6000. Read alignment, quality control, differential expression analysis, and pathway analysis was performed by Novogene using the standard pipeline or GENAVi (84). CIBERSORT analysis was performed by TIMER2.0 using the immune estimation function (http://timer.cistrome.org/). Gene set enrichment analysis (GSEA; v4.2.3) was performed on DESeq2-normalized counts using the gene_set permutation type with Mammary stem/progenitor signatures (28) accessed via the MsigDB and previously published late recurrence or late distant metastasis signatures (30).

### Clinical analysis

Survival and expression correlation analysis of select genes was performed using the Kaplan-Meier Plotter tool (breast cancer, mRNA gene chip; https://kmplot.com/). Expression levels of *Dkk3* (Affymetrix ID 214247_s_at) were split by upper and lower quartiles to compare relapse-free survival (RFS) by PAM50 subtype. Overall survival (OS) data, PAM50 classification, and RNA-seq log_2_-normalized counts for the TCGA-BRCA data set were accessed via the UCSC Xena Browser (https://xenabrowser.net/) and stratified based on upper and lower *Dkk3* quartiles for survival. METABRIC and the Metastatic Breast Cancer Project (MBCProject) mRNA expression data and clinical characteristics were accessed via cBioPortal (https://www.cbioportal.org/).

### Statistics

No statistical methods were used to predetermine animal numbers and when appropriate animals were randomly assigned to treatment groups using the RANDBETWEEN function in Microsoft Excel. Data were visualized and analyzed using GraphPad Prism v9, with each point representing a single mouse for in vivo experiments or technical replicates for in vitro experiments. In vitro experiments were performed at least twice with similar results and in vivo experiments were repeated with similar results or validated with complementary experiments. Details of statistical analyses can be found in the figure legends and *P* values are displayed within the figures. *P* values of 0.05 or less were considered significant.

### Study approval

All animal studies were performed in accordance with Duke IACUC approval (protocol A043-23-02) and supervised/housed by the Division of Laboratory Animal Resources (DLAR).

### Data availability

The tumor and cell line bulk RNA-seq data have been submitted to the NCBI Gene Expression Omnibus (GEO) database (GSE240021). Individual data values for the main figures and supplement can be found in the Supporting Data Values file.

## Author contributions

TNT, HKL, and ZCH conceptualized and acquired funding for the project. TNT and ZCH designed experiments and wrote the manuscript. TNT performed experiments with assistance from CED and DS and analyzed/interpreted the data. TW provided technical support for flow cytometry and XY provided support for tissue processing and sectioning. CRA provided bioinformatics advice. ZCH generated lentiviral vectors with assistance from JW and GL.

## Supplementary Material

Supplemental data

Supporting data values

## Version 1 10/17/2023

In-Press Preview

## Version 2 11/22/2023

Electronic publication
