# Supplementary material for: Dormant tumors circumvent tumor-specific adaptive immunity by establishing a Treg-dominated niche via DKK3
Source: JCI Insight. 2023 Nov 22;8(22):e174458. doi: 10.1172/jci.insight.174458 (PMC10721325; doi:10.1172/jci.insight.174458)
Supplement: Supplemental data [file jciinsight-8-174458-s151.pdf]

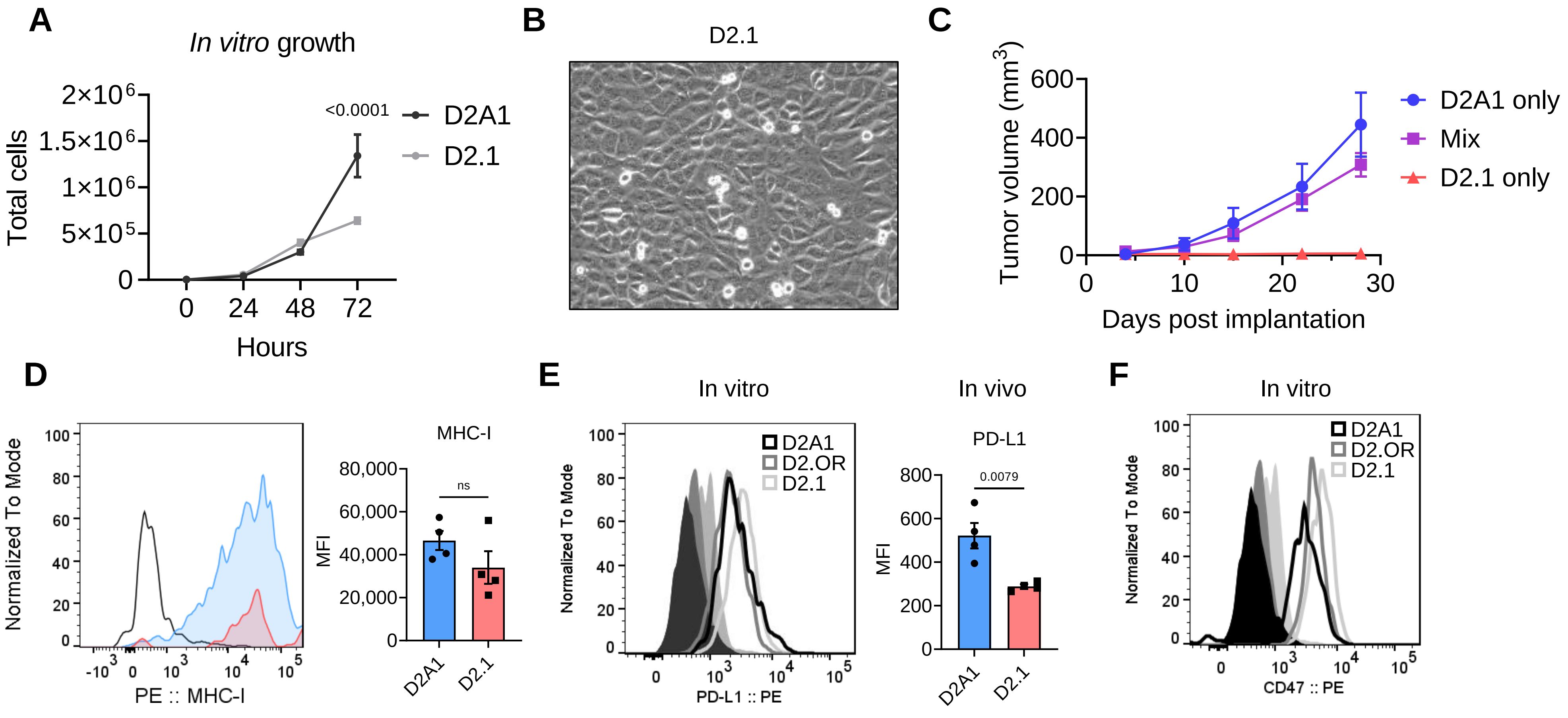

Supplementary figure 1:

A In vitro growth of D2A1 and D2.1 cells. Statistical comparison by two way ANOVA. **B** Phase contrast image of D2.1 cells at confluence. **C** Growth of 10^6 D2A1 (n=3), 10^6 D2.1 (n=3), or a mix of 5×10^5 D2A1 and 5×10^5 D2.1 cells (n=3) implanted into the mammary fat pad of female Balb/c mice. **D** Representative flow plot and MFI of D2A1 (n=4) or D2.1 (n=4) tumors after 35 days in mice. **E** PD-L1 surface expression on D2A1, D2.OR, and D2.1 cells in vitro (left) or D2A1 or D2.1 tumors from D. **F** CD47 expression of D2A1, D2.OR, and D2.1 cells in vitro. Comparisons were performed by two-tailed ttest (D,E). Error bars represent mean ± SEM.

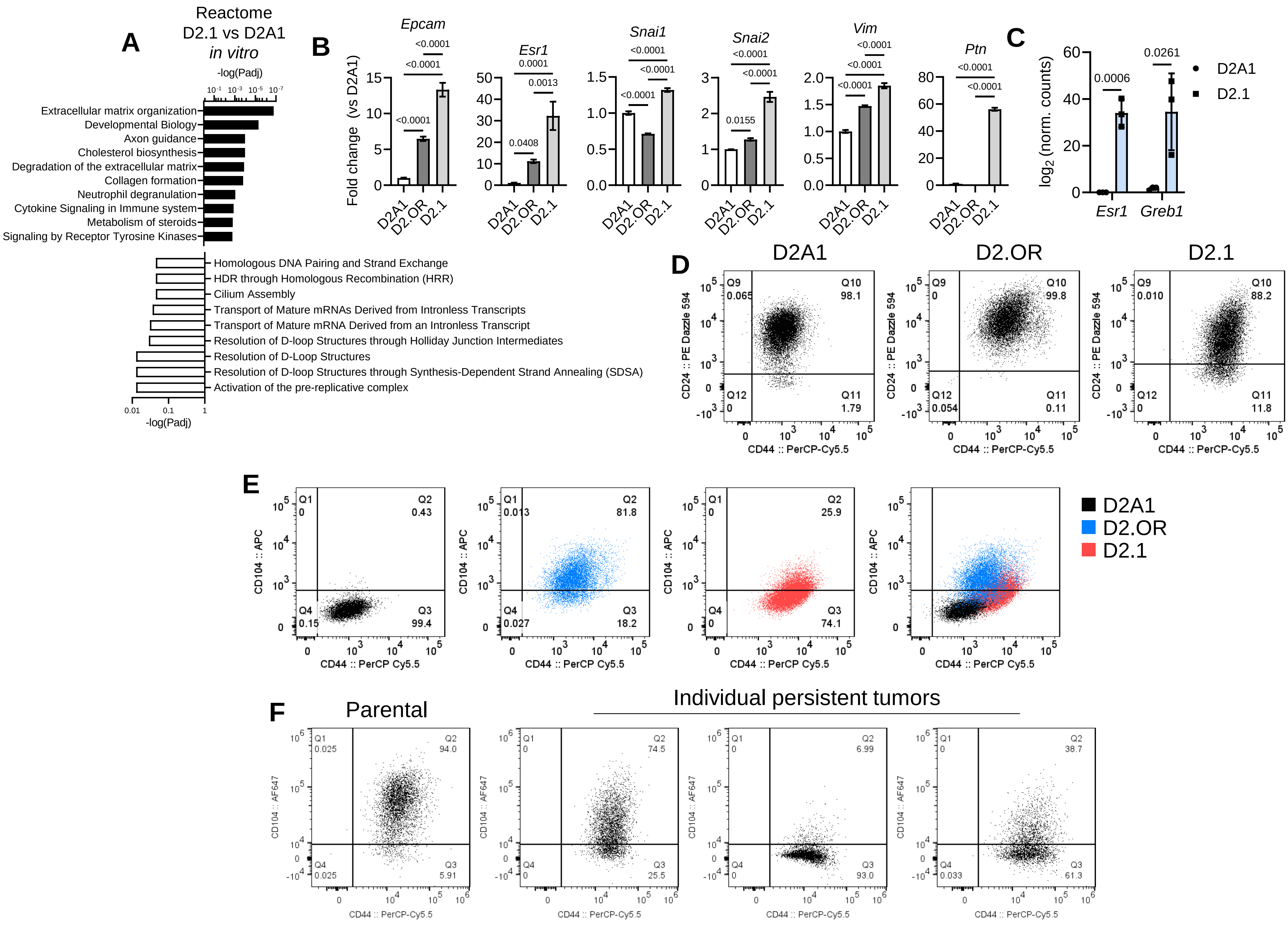

Supplementary figure 2:

A Reactome analysis of D2.1 or D2A1 cells related to Figure 5A. Displayed are the top ten upregulated (black fill) and downregulated (white fill) pathways. **B** Quantitative PCR in parental D2A1, D2.OR, and D2.1 cells of select genes associated with Figure 2B. Error bars represent mean ± SD. P values were determined by one-way ANOVA with Tukey's post-hoc correction. **C** *Esr1* and *Greb1* gene expression from RNAseq of D2.1 or D2A1 cells. Error bars represent mean ± SD. P values between D2.1 and D2A1 were determined by two-tailed ttest. **D** Representative flow plots of surface staining for CD24 and CD44 in cultured D2A1, D2.OR, and D2.1 cells. **E** Staining of CD104 and CD44 in cultured D2 cells. **F** Surface staining for CD104 and CD44 of D2.OR parental cells (left) or independent populations of surviving, residual D2.OR nodules recovered over 100 days after implantation into the MFP of Balb/c mice (right).

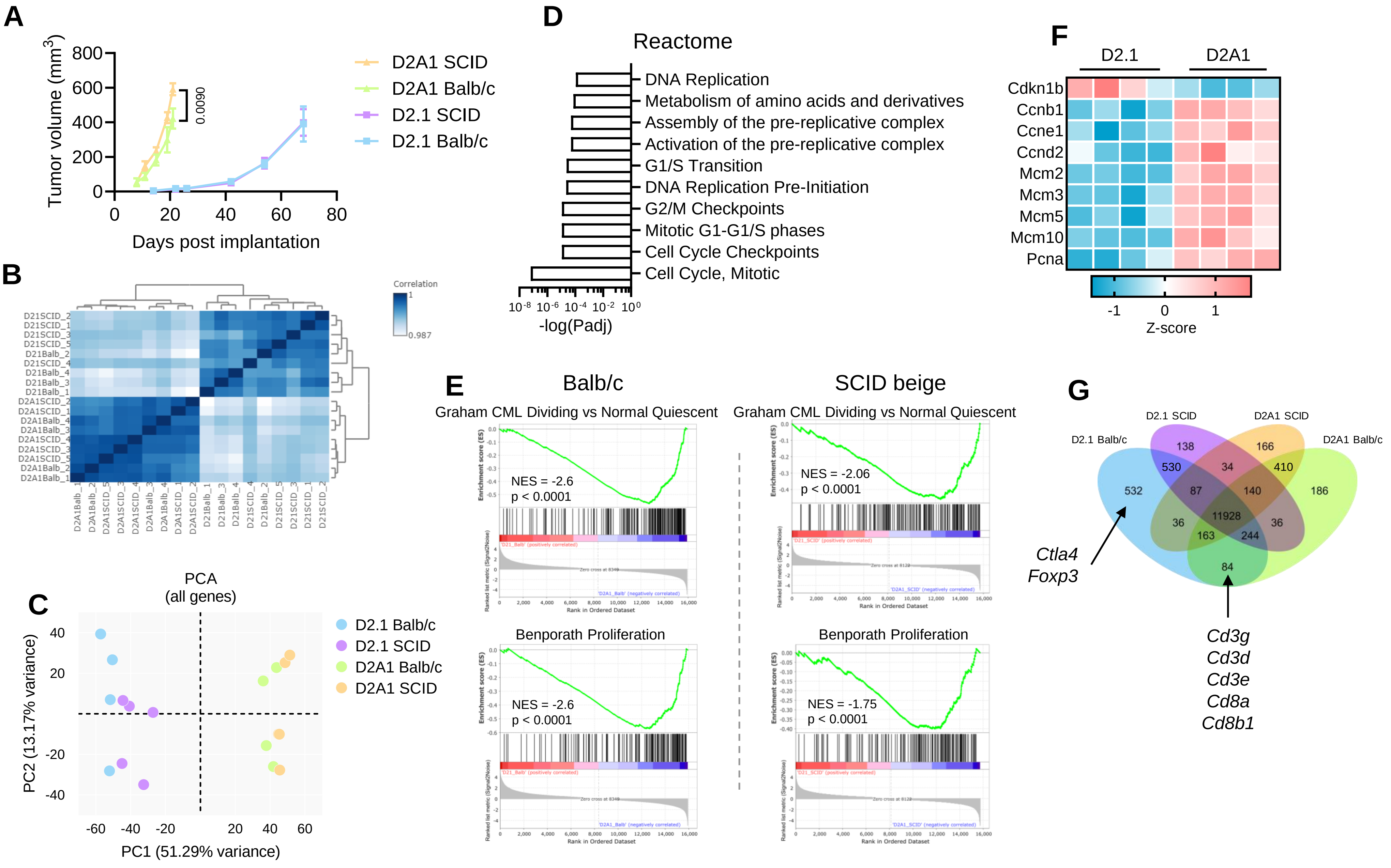

Supplementary figure 3:

A Growth of 10^6 D2A1 or D2.1 cells implanted into the MFP of female Balb/c or SCID beige mice related to Figure 2. Comparison shown is by two way ANOVA at time of collection for sequencing. **B** Correlation of RNA-seq data of D2.1 and D2A1 tumors from A. **C** Principal component analysis (PCA) of RNA-seq from tumors in A. **D** Ten most downregulated Reactome pathways in Balb/c D2.1 tumors compared to Balb/c D2A1. **E** Proliferation related GSEA enrichment plots in D2.1 vs D2A1 tumors in Balb/c (left) or SCID beige (right) mice. **F** Heatmap of differentially expressed dormancy-related genes in Balb/c D2.1 tumors compared to D2A1. **G** Venn diagram of tumors from A with annotated genes.

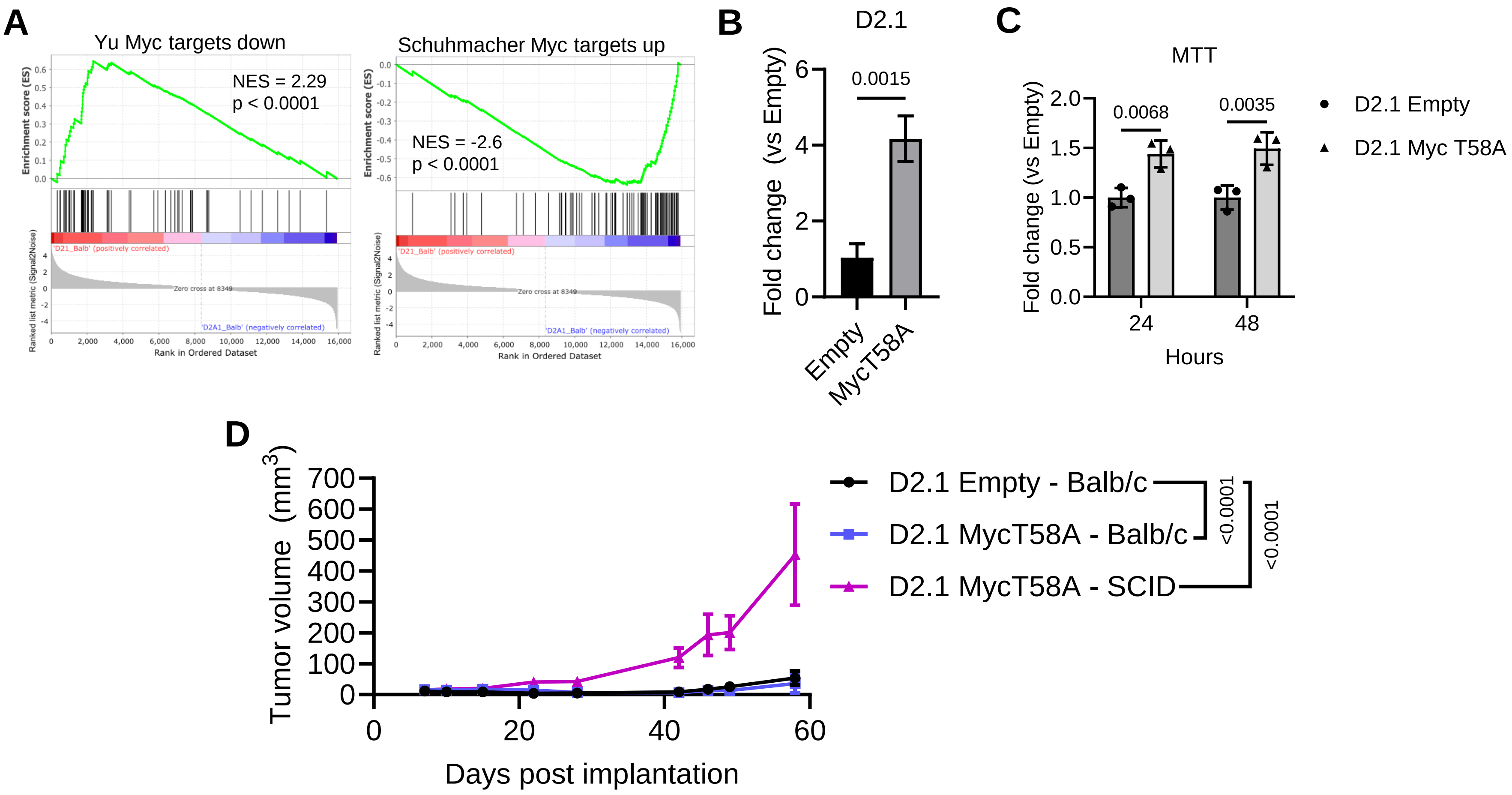

Supplementary figure 4:

A GSEA of Balb/c D2.1 vs D2A1 tumors related to Myc signaling. **B** Quantitative PCR analysis of Empty vector control or D2.1 cells engineered to overexpress MycT58A. Groups were compared by two-tailed ttest and error bars represent mean ± SD. **C** In vitro growth of cells from A. Error bars represent mean ± SEM and comparisons were performed by Šídák two-way ANOVA. **D** Growth of 10^6 D2.1 cells overexpressing MycT58A or Empty vector control cells in the MFP of female Balb/c of SCID beige mice. Comparisons shown are by two way ANOVA with Tukey’s post hoc correction and error bars represent mean ± SEM.

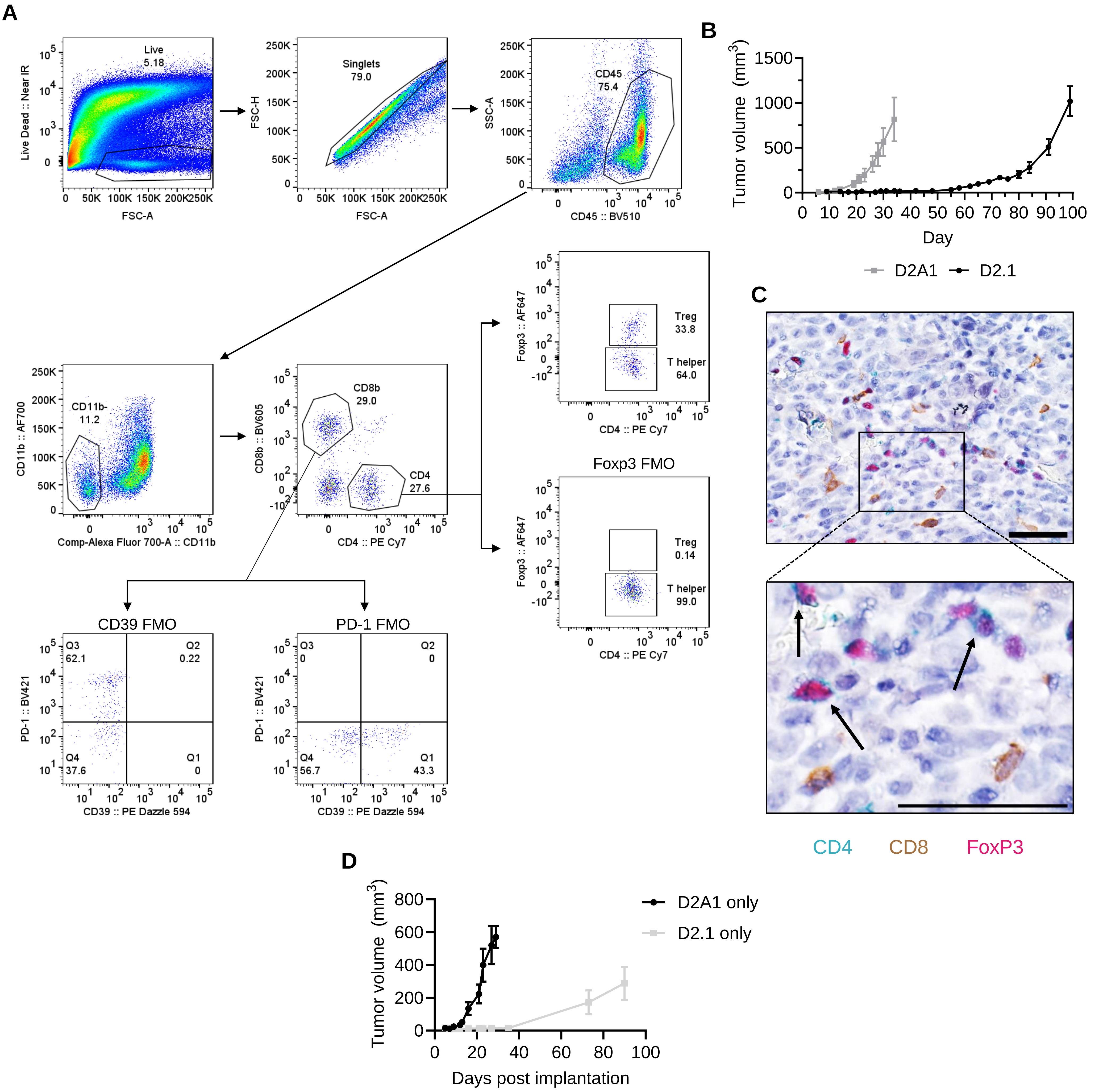

Supplementary figure 5:

A Representative gating strategy (from tumor) used to identify T cell populations. The same gating strategy was used for tumors and spleens in vivo as well as subsequent in vitro experiments. **B** Tumor growth plot associated with Figure 3H-L. **C** Triple IHC for CD4, CD8, and FoxP3 in D2.1 tumor. Arrows indicate double labeled CD4+FoxP3+ cells and scale bars represent 25 µm. **D** Overall tumor growth plots of tumors from 4A. Error bars represent mean ± SEM.

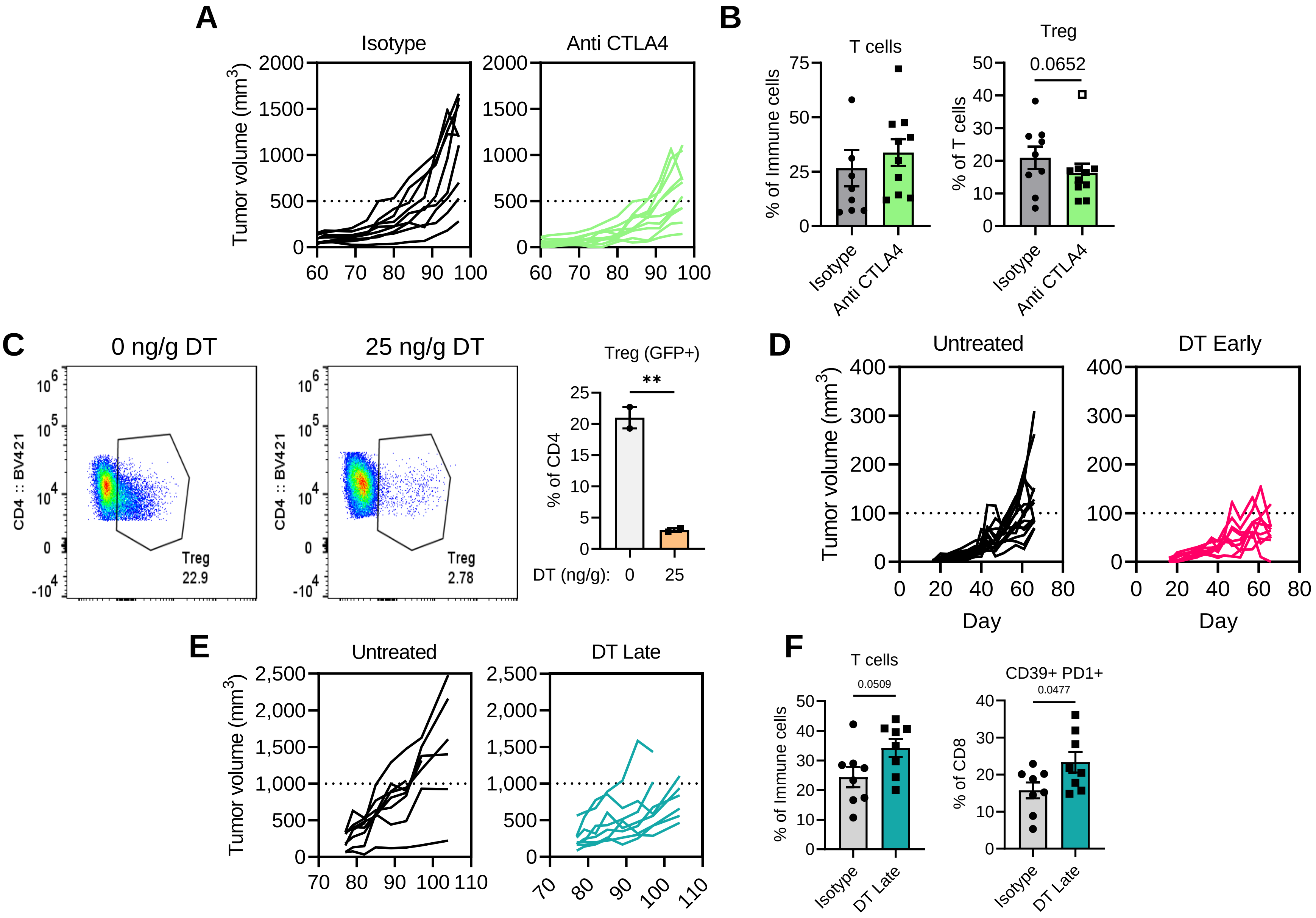

Supplementary figure 6:

A Individual growth of tumors from Figure 5H. **B** T cell profiles of tumors from A. An outlier was identified (right; empty square) using the outlier analysis function in GraphPad Prism (ROUT method, q = 1%) and the P value displayed excludes the outlier (p = 0.3173 included). **C** Representative flow plot (left) and individual values (right) of Tregs in spleens 2 days post final DT (25 ng/g) treatment in naïve animals. **D, E** Growth of individual tumors from Figure 5J. **F** CD8 CD39+ PD-1+ cells in Untreated or DT late tumors from E. Statistical comparisons of two groups were performed by two-tailed ttest and error bars represent mean ± SEM.

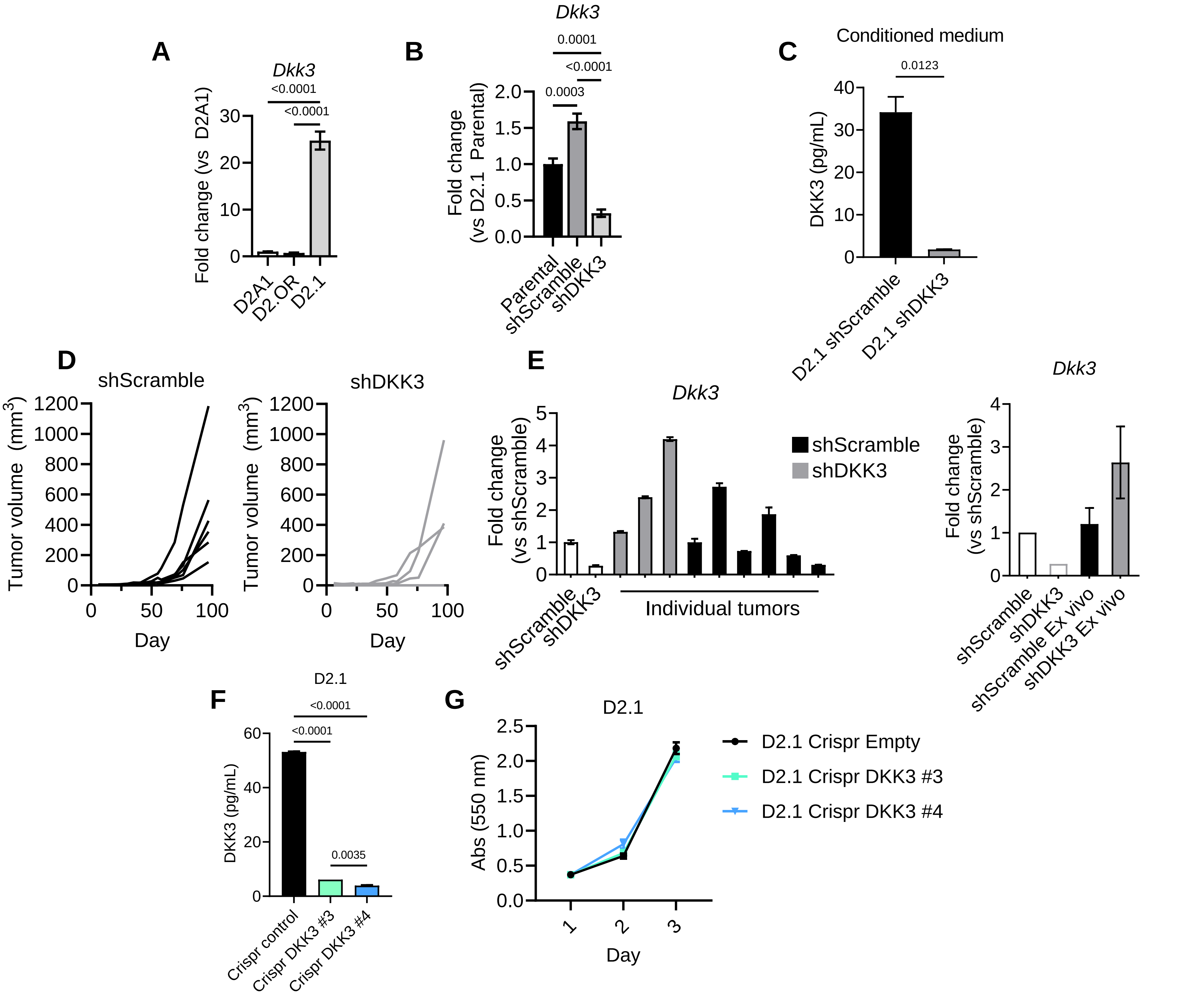

Supplementary figure 7:

A Quantitative PCR analysis of *Dkk3* mRNA expression in parental D2A1, D2.OR, and D2.1 cells. **B** Analysis of *Dkk3* mRNA in parental, shScramble control, or shDKK3 D2.1 cells by quantitative PCR. **C** ELISA for DKK3 protein on CM from D2.1 shScramble or shDKK3 cells. **D** Individual D2.1 tumor growth related to Figure 6C. **E** Quantitative PCR analysis of *Dkk3* mRNA expression after tumors from D were digested and plated to generate pure tumor populations ex vivo. Left panel is individual tumors and right panel shows the mean of each group. **F** ELISA for DKK3 protein in CM from D2.1 non-targeting Crispr control cells or two independent guide RNAs directed towards *Dkk3*. **G** Growth of D2.1 cells from F. Comparisons of two groups were performed by two-tailed ttest (C) and three groups were performed by one-way ANOVA with Tukey's post-hoc analysis (A, B, F). Error bars represent mean ± SEM.

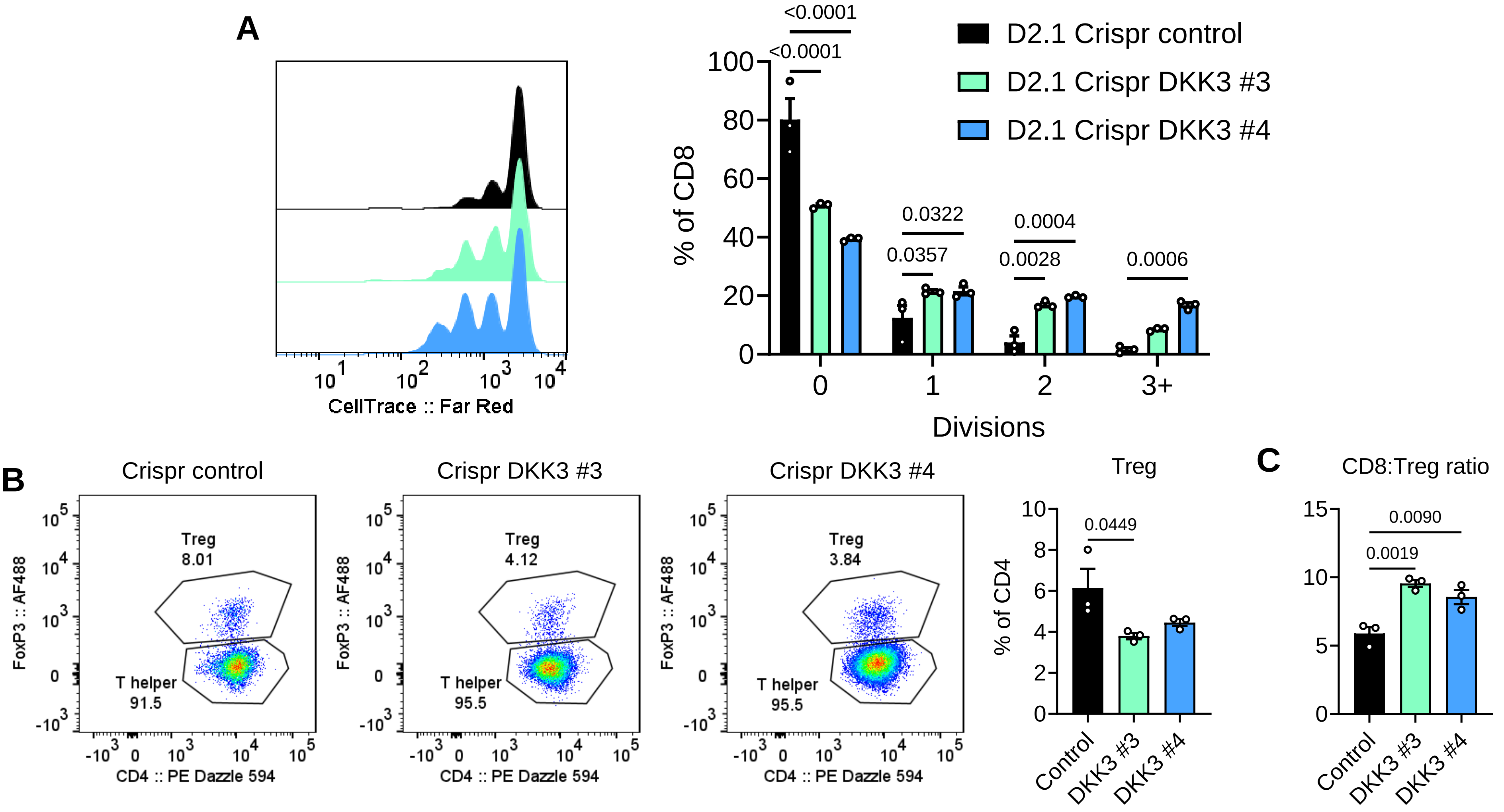

Supplementary figure 8:

A Representative flow plot (left) and quantification of CD8 cell divisions after 3 days when whole Jedi splenocytes were cultured in D2.1 Crispr control or Crispr DKK3 CM with anti-CD28 antibodies and eGFP$_{200\text{-}208}$ peptide. Statistical comparisons were performed using two-way ANOVA with Dunnett's multiple comparisons test. **B** Quantification of Tregs in CD4 cells from A. **C** Ratio of CD8 cells to Tregs in cultures from A. Comparisons for B, C were performed by one-way ANOVA with Tukey's post-hoc analysis. Error bars represent mean ± SEM.

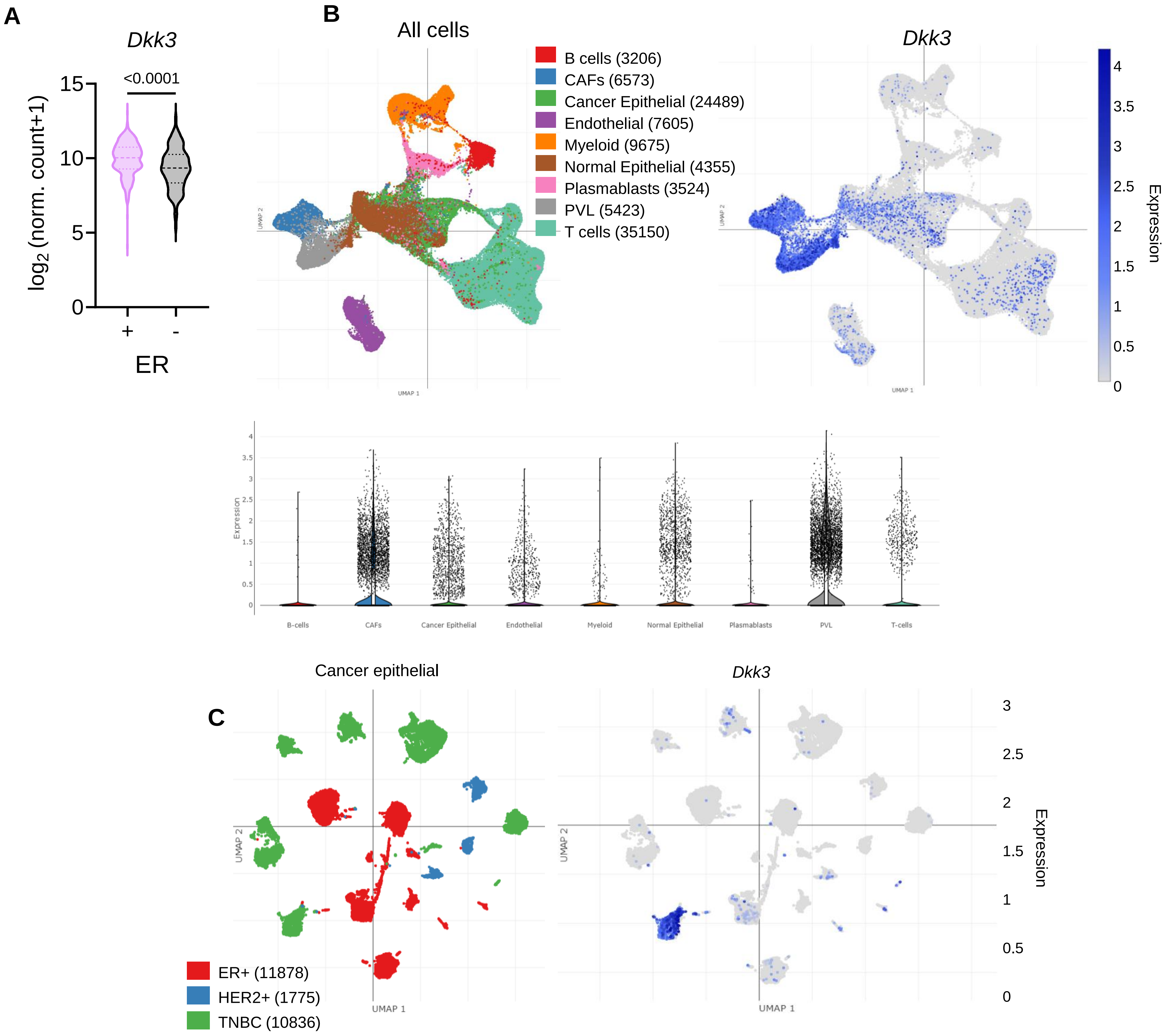

Supplementary figure 9:

A *Dkk3* expression by ER positivity in TCGA BC samples. Comparisons were performed by or by ttest. **B** Single-cell RNA-sequencing of 26 human breast tumor samples accessed via the Broad Institute single-cell portal (https://singlecell.broadinstitute). *Dkk3* expression is shown both overlaid on UMAP plot and as individual expression values grouped by annotated cell type. **C** *Dkk3* expression overlaid on UMAP plot of cancer epithelial cells of ER+, HER2+, or TNBC after sub-clustering. Plots can be accessed via https://singlecell.broadinstitute.org/single_cell/study/SCP1039/a-single-cell-and-spatially-resolved-atlas-of-human-breast-cancers?genes=DKK3&cluster=Epithelial%20cells&spatialGroups=--&annotation=subtype--group--study&subsample=all&tab=scatter&scatterColor=Blues&distributionPlot=violin&distributionPoints=all#study-visualize

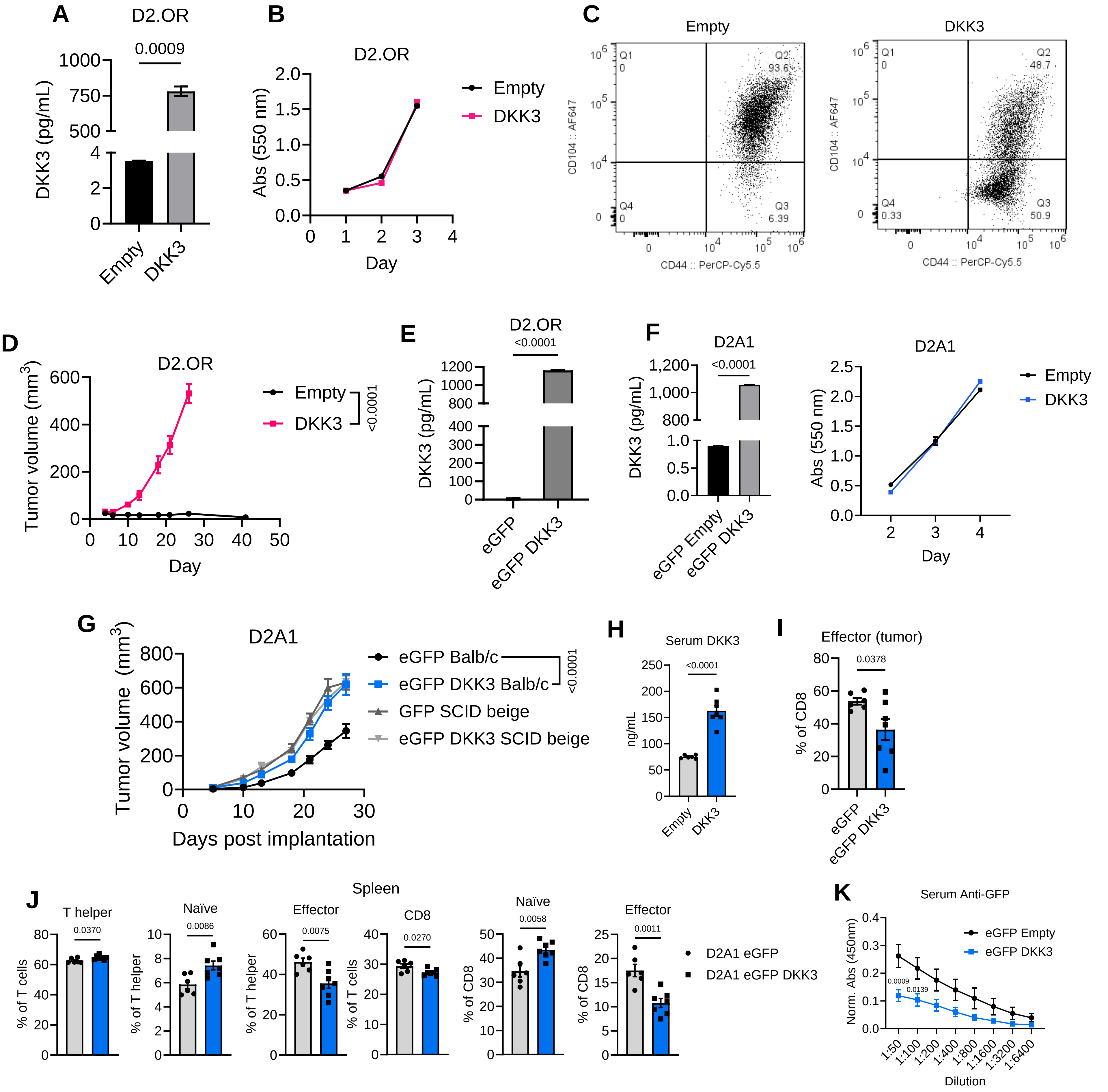

Supplementary figure 10:

A ELISA for DKK3 in CM from D2.OR cells engineered to express DKK3 or Empty control. **B** Growth of cells in culture from A. **C** Surface expression of CD44 and CD104 in D2.OR cells from A expressing DKK3 or Empty control. **D** Growth of 10^6 D2.OR DKK3 or Empty control cells in the MFP of female Balb/c mice with p value at time of D2.OR DKK3 euthanasia by two way ANOVA shown. **E** ELISA on CM from D2.OR eGFP only or D2.OR eGFP DKK3 cells. **F** ELISA for DKK3 protein on CM (left) and growth in vitro (right) of D2A1 eGFP Empty control or eGFP DKK3 expressing cells. **G** Growth of control or D2A1 eGFP DKK3 tumors (10^6 cells) in the MFP of female Balb/c or SCID beige mice with comparison by Šídák's two-way ANOVA at end of experiment shown. **H** Serum DKK3 levels by ELISA at time of euthanasia from G. **I** Effector CD8 T cells in tumors of mice from G. **J** T cell phenotyping in spleens of Balb/c mice from G. Effector cells were defined as CD44+ CD62l- and Naïve cells were defined as CD44- CD62l+. **K** Anti-eGFP antibodies in serum of Balb/c mice from G normalized by tumor volume and compared by two-way ANOVA. Statistical analysis of two groups (A, E, F, H, I, J) was performed by two-tailed ttest. Error bars represent mean ± SEM.

Cheng_Late recurrence

SKAP2	FAM65A
LIMS1	COL6A3
PLXDC1	THY1
MAFB	PKD2
SHOX2	NID2
LOH3CR2A	VCAN
NNMT	TGFB1I1
TNC	CALM1
MRC2	CXCR7
LAMA4	IL13RA1
DYRK2	COL1A2
SEMA5A	THY1
SHOX2	PDE8A
SNAI2	ATP2B1
SEC23A	ITGA5
GNS	RABGGTA
HTRA1	LRRC32
DDEF1IT1	CDKN1A
LAMB1	POSTN
COL5A1	TWIST1
JAG1	ATP2B1
COL1A1	M6PRBP1
EMILIN1	ADAM9
PHTF2	DYRK2
COL5A1	HSPG2
CLEC11A	TPP1
JAG1	AEBP1
ANGPTL2	MRPL52
COL3A1	

Mittempergher_Late distant metastasis

CXCL13	IBSP	DPT	CCR6	IGHA1	ZNF224
CREG2	TFF1	ESR1	CD36	BACH1	LOC51152
LOC348174	STAC	CDC14A	IGHA1	IGHV1-69	NRG1
IRF4	LOC155006	SPINK4	GRAP2	TUB	CEACAM1
SYT13	WFDC2	BRUNOL6	ARMC4	COLEC11	HIST1H1D
IGJ	ADD3	BAMBI	LOC131873	CDC42EP3	GNG2
IGHM	PKIA	WBSCR28	IGHA1	AIM2	IGLV6-57
IGF1	LOC652791	RFPL1S	FLJ39660	CEACAM5	TMED6
CBLN2	WISP2	LPHN3	MAP9	ADAMTS19	CCDC65
LMO4	SLC39A6	TFAP2B	HS6ST2	CCL19	ITM2A
DIO3	KLRC1	IL7	C7	CTNND2	
SLC16A7	SFTPA1	LOC439949	FREM1	CFHR1	
CH25H	OMD	KLF5	SLC40A1	CXCL9	
SELE	OGN	PTGER4	GJB6	KIAA1822L	
PITX2	LOC644488	CPB1	XCL1	LOC652159	
BAAT	TFF1	C8ORFK32	WNT10A	TMEM119	
TNFSF15	C21orf34	KLRB1	PPP2R2C	VGLL3	
BHLHB5	NOPE	MGAT5B	DSCR6	POU2AF1	
FABP4	MYCBPAP	CA14	FLJ39370	LAX1	
LUZP2	CFH	ZBTB16	C10orf30	SPFH2	
KLK12	CACNA2D2	CD96	CDO1	CSMD2	
IGHA1	TNN	HP	LOC645733	ASPN	
TNFRSF17	ADD3	SLC23A1	IGHA1	CNTN4	
CTNND2	LOC440361	MFAP4	XG	ZBTB12	
SLC39A6	CA3	LOC390712	ABCA6	IGF1	
WFDC2	HIST1H2AL	GREM2	TSPAN13	WASF3	
GRIA2	NRG2	NBPF4	HAVCR1	SH2D1A	
MAP9	MMP3	ANK1	NELL2	KCNB1	
GALNT3	RASSF8	LOC644846	IGKV1-5	PVRL3	

Supplementary table 1: Gene lists used for GSEA analysis in reference to Fig. 2.

Dkk1

	Pearson Correlation				p value		
	TCGA	KMplot	Metabric		Metabric	TCGA	KMplot
Cd4	0.0264	-0.0233	-0.1160	Cd4	0.0943	0.7966	0.4717
Cd8a	-0.0136	-0.0569	-0.0679	Cd8a	0.3288	0.8947	0.0793
Cd8b	-0.0654	-0.0429	0.0875	Cd8b	0.2079	0.5226	0.1858
Gzma	0.0416	-0.0425	-0.0330	Gzma	0.6351	0.6843	0.1900
Gzmb	0.05114	-0.0375	-0.0736	Gzmb	0.2897	0.6170	0.2481
Prf1	0.0677	-0.0565	-0.0919	Prf1	0.1855	0.5078	0.0815
Ifng	0.0315	-0.0389	-0.0957	Ifng	0.1680	0.7580	0.2303
Tnf	0.1736	0.0911	0.0347	Tnf	0.6179	0.0873	0.0049
Entpd1	0.1020	-0.0460	0.0364	Entpd1	0.6009	0.3175	0.1560
Nt5e	-0.0293	-0.0541	0.0199	Nt5e	0.7754	0.7745	0.1225
Mki57	0.0366	0.1186	-0.1370	Mki57	0.0479	0.7203	0.0002

Dkk2

	Pearson Correlation				p value		
	TCGA	KMplot	Metabric		Metabric	TCGA	KMplot
Cd4	0.4359	0.2434	0.1640	Cd4	0.0176	0.0000	<1E-04
Cd8a	0.3497	0.1844	0.1954	Cd8a	0.0046	0.0004	<1E-04
Cd8b	0.1597	0.1516	0.0406	Cd8b	0.5594	0.1162	<1E-04
Gzma	0.3253	0.1466	0.1696	Gzma	0.0141	0.0011	<1E-04
Gzmb	0.2383	0.1155	0.1921	Gzmb	0.0053	0.0182	0.0004
Prf1	0.2595	0.0993	0.1281	Prf1	0.0646	0.0099	0.0021
Ifng	0.1952	0.0986	0.1806	Ifng	0.0089	0.0541	0.0023
Tnf	0.0421	0.0413	-0.0340	Tnf	0.6253	0.6805	0.2022
Entpd1	0.4891	0.2942	-0.0080	Entpd1	0.9085	0.0000	<1E-04
Nt5e	0.1788	0.1237	0.0766	Nt5e	0.2704	0.0782	0.0001
Mki67	-0.1539	-0.1074	0.0005	Mki67	0.9944	0.1303	0.0009

Dkk3

	Pearson Correlation				p value		
	TCGA	KMplot	Metabric		Metabric	TCGA	KMplot
Cd4	-0.0082	0.0017	-0.2566	Cd4	0.0002	0.9359	0.9581
Cd8a	-0.1066	-0.1632	-0.2247	Cd8a	0.0011	0.2961	<1E-04
Cd8b	-0.0662	-0.0433	-0.1396	Cd8b	0.0438	0.5174	0.1817
Gzma	-0.1059	-0.0570	-0.2086	Gzma	0.0024	0.2992	0.0784
Gzmb	-0.2505	-0.0585	-0.3222	Gzmb	<0.0001	0.0129	0.0711
Prf1	-0.1250	-0.1009	-0.3026	Prf1	<0.0001	0.2203	0.0018
Ifng	-0.2758	-0.2795	-0.2937	Ifng	<0.0001	0.0060	<1E-04
Tnf	-0.0804	-0.1813	-0.2160	Tnf	0.0017	0.4313	<1E-04
Entpd1	0.1883	0.1701	0.2799	Entpd1	<0.0001	0.0634	<1E-04
Nt5e	0.5004	0.2883	0.3305	Nt5e	<0.0001	0.0000	<1E-04
Mki67	-0.1998	-0.1454	-0.4799	Mki67	<0.0001	0.0486	<1E-04

Supplementary table 2: Individual Pearson Correlation (2 tailed) and p values in reference to Fig. 7c.

Cell line	Derived from	Strain	Syngeneic	Dormant?	Immune sensitive?	EMT status	ER+
D2A1	D2HAN (D2-type hyperplastic alveolar nodules) [14]	Balb/c	Yes	No	Medium	Mesenchymal	No
D2.OR		Balb/c	Yes	Yes (lungs[16])	High	Hybrid	Yes
D2.1		Balb/c	Yes	Yes (fat pad, lungs[15,16])	Low	Hybrid	Yes

Supplementary table 3: Overall phenotype of D2 cells as described herein and by others.

Target	Conjugate	Clone	Catalog #	Vendor	Use
CD104	APC	346-11A	123611	BioLegend	Flow cytometry
CD104	AF647	346-11A	123607	BioLegend	Flow cytometry
CD11b	PerCP Cy5.5	M1/70	101227	BioLegend	Flow cytometry
CD11b	AF700	M1/70	101222	BioLegend	Flow cytometry
CD24	PE Dazzle 594	M1/69	101837	BioLegend	Flow cytometry
CD39	PE Dazzle 594	Duha59	143811	BioLegend	Flow cytometry
CD4	Unconjugated	D7D2Z	25229	Cell Signaling	IHC
CD4	PE Dazzle 594	RM4-5	100566	BioLegend	Flow cytometry
CD4	BV421	RM4-5	100543	BioLegend	Flow cytometry
CD44	PerCP Cy5.5	IM7	103032	BioLegend	Flow cytometry
CD45	BV605	30-F11	103140	BioLegend	Flow cytometry
CD47	PE	miap301	127507	BioLegend	Flow cytometry
CD62L	PE	MEL-14	104407	BioLegend	Flow cytometry
CD8b	APC Cy7	YTS156.7.7	126620	BioLegend	Flow cytometry
CD8b	PE Cy7	YTS156.7.7	126616	BioLegend	Flow cytometry
CD8b	BV605	H35-17.2	740387	BD Biosciences	Flow cytometry
CD8α	Unconjugated	D4W2Z	98941	Cell Signaling	IHC
GFP	Unconjugated	Polyclonal	GFP-1020	Aves Labs	IF
FoxP3	Unconjugated	D6O8R	12653S	Cell Signaling	IHC
FoxP3	AF488	MF-14	126406	BioLegend	Flow cytometry
FoxP3	Unconjugated	FJK-16s	14-5773-82	Invitrogen	IHC
Ki-67	Unconjugated	Polyclonal	NB110-89717SS	Novus Biologicals	IF
MHC I H2Kd/H2Dd	PE	34-1-2S	114713	BioLegend	Flow cytometry
PD-1 (CD279)	BV421	29F.1A12	135218	BioLegend	Flow cytometry
PD-L1 (CD274)	PE	10F.9G2	124307	BioLegend	Flow cytometry

Supplementary table 4: Antibodies used for staining.

Supplementary Methods

Anti-GFP ELISA

Immulon 4 HBX (Thermo Scientific) plates were coated with 1 µg/mL recombinant A. victoria GFP (Abcam) at 4°C. The following day plates were washed with PBS + 0.05% Tween 20 and blocked with PBS + 1% BSA (Sigma) for 1 hour at 37°C. A serial dilution (in 1% BSA/PBS) of serum was added in duplicate for 2 hours at room temperature followed by anti-mouse IgG streptavidin-HRP conjugated antibody (1:2000 in 1% BSA/PBS; Cell Signaling Technology) for 1 hour at 37°C. Plates were developed with TMB substrate (BioLegend), stopped with 0.18 M H_2SO_4, and read at 450 nm on a Bio-Rad 680 microplate reader.
